# Mechanistic insights into tRNA cleavage by a contact-dependent growth inhibitor protein and translation factors

**DOI:** 10.1093/nar/gkac228

**Published:** 2022-04-12

**Authors:** Jing Wang, Yuka Yashiro, Yuriko Sakaguchi, Tsutomu Suzuki, Kozo Tomita

**Affiliations:** Department of Computational Biology and Medical Sciences, Graduate School of Frontier Sciences, The University of Tokyo, Kashiwa,Chiba277-8562, Japan; Department of Computational Biology and Medical Sciences, Graduate School of Frontier Sciences, The University of Tokyo, Kashiwa,Chiba277-8562, Japan; Department of Chemistry and Biotechnology, Graduate School of Engineering, The University of Tokyo, Bunkyo-ku, Tokyo 113-8656, Japan; Department of Chemistry and Biotechnology, Graduate School of Engineering, The University of Tokyo, Bunkyo-ku, Tokyo 113-8656, Japan; Department of Computational Biology and Medical Sciences, Graduate School of Frontier Sciences, The University of Tokyo, Kashiwa,Chiba277-8562, Japan

## Abstract

Contact-dependent growth inhibition is a mechanism of interbacterial competition mediated by delivery of the C-terminal toxin domain of CdiA protein (CdiA–CT) into neighboring bacteria. The CdiA–CT of enterohemorrhagic *Escherichia coli* EC869 (CdiA–CT^EC869^) cleaves the 3′-acceptor regions of specific tRNAs in a reaction that requires the translation factors Tu/Ts and GTP. Here, we show that CdiA–CT^EC869^ has an intrinsic ability to recognize a specific sequence in substrate tRNAs, and Tu:Ts complex promotes tRNA cleavage by CdiA–CT^EC869^. Uncharged and aminoacylated tRNAs (aa-tRNAs) were cleaved by CdiA–CT^EC869^ to the same extent in the presence of Tu/Ts, and the CdiA–CT^EC869^:Tu:Ts:tRNA(aa-tRNA) complex formed in the presence of GTP. CdiA–CT^EC869^ interacts with domain II of Tu, thereby preventing the 3′-moiety of tRNA to bind to Tu as in canonical Tu:GTP:aa-tRNA complexes. Superimposition of the Tu:GTP:aa-tRNA structure onto the CdiA–CT^EC869^:Tu structure suggests that the 3′-portion of tRNA relocates into the CdiA–CT^EC869^ active site, located on the opposite side to the CdiA–CT^EC869^ :Tu interface, for tRNA cleavage. Thus, CdiA–CT^EC869^ is recruited to Tu:GTP:Ts, and CdiA–CT:Tu:GTP:Ts recognizes substrate tRNAs and cleaves them. Tu:GTP:Ts serves as a reaction scaffold that increases the affinity of CdiA–CT^EC869^ for substrate tRNAs and induces a structural change of tRNAs for efficient cleavage by CdiA–CT^EC869^.

## Graphical Abstract

**Graphical Abstract:**
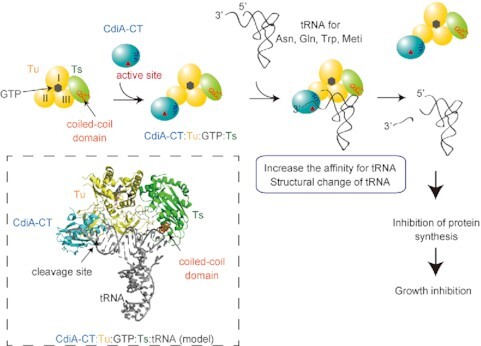
Model of tRNA cleavage by CdiA–CT in the presence of translation factors Tu/Ts and GTP. The Tu:GTP:Ts complex acts as a scaffold for tRNA cleavage by CdiA–CT.

## INTRODUCTION

The contact-dependent growth inhibition (CDI) system is a mechanism of interbacterial competition that is widely observed in Gram-negative bacteria and common in pathogenetic proteobacteria (1–5). CDI requires direct physical contact between bacteria and is mediated by specific receptors and the delivery of toxin proteins. Toxin delivery into neighboring bacteria induces growth inhibition and is occasionally fatal. In *Escherichia coli*, the genes responsible for CDI are *cdiA*, *cdiB* and *cdiI* (1). CdiA, which adopts a large filamentous structure, is attached to the cell surface via its N-terminus and contains a receptor-binding domain (RBD) and a C-terminal toxin domain (CdiA–CT) (4,6,7). CdiB is an outer membrane protein through which CdiA is secreted. When the RBD of CdiA binds to the receptor of neighboring bacteria, CdiA autoproteolytically cleaves the CdiA–CT, which then translocates into the target cells. CdiI is an immunity protein that protects cells from self-intoxication by forming a tight CdiA–CT:CdiI complex (8,9). Accordingly, the growth of non-isogenic bacteria without the corresponding CdiI is inhibited by CdiA–CT. CdiA–CT toxins are highly polymorphic and include various types of toxin modules with pore-forming potential, RNase activity targeting the 16S rRNA or tRNA (tRNase), or DNase activity. Most *E. coli* CdiA–CT toxins have nuclease activity (2).

Cytosolic factors are sometimes required for the activity of CdiA–CT toxin (10–16). CdiA toxin from *E. coli* 563, CdiA–CT^EC563^, is a potent tRNA anticodon nuclease (1,10) that requires *o*-acetylserine sulfhydrylase A (CysK) for its RNase activity *in vivo* and *in vitro* (10,12,13). CysK interacts with the C-terminal Gly-Tyr-Gly-Ile motif of CdiA–CT^EC563^ and increases the tRNase activity of CdiA–CT^EC563^ by increasing its thermostability and promoting interactions with substrate tRNAs (10,11,15). Recently, it was shown that CdiAs from *E. coli* NC101 (CdiA–CT^NC101^), EC869 (CdiA–CT^EC869^), and *Klebsiella pneumoniae* 342 (CdiA–CT^Kp342^) all cleave the 3′ regions of the acceptor stems of specific tRNAs (14–17). CdiA–CT^EC869^ cleaves the 3′-acceptor stems of tRNA^Gln^ and tRNA^Asn^ between positions 71 and 72 (15); CdiA–CT^NC101^ cleaves tRNA^Glu^, tRNA^Asp^ and others between positions 72 and 73 (14); and CdiA–CT^Kp342^ cleaves tRNA^Ile^ (16). *In vitro*, EF-Tu and EF-Ts (hereafter simply Tu and Ts, respectively) together with GTP promote cleavage of tRNA by these CdiA–CT toxins. *In vivo*, *tsf* mutants in the coiled-coil domain of Ts are resistant to inhibition by these CdiA–CTs (14–16). Although CdiA–CT^NC101^ and CdiA–CT^Kp342^ exhibit low similarity at the primary amino acid sequence level, their structures are homologous and adopt the Barnase/EndoU/Colicin/RelE (BECR) fold, suggesting that the similar structures of CdiA–CTs arose through convergent evolution (16). On the other hand, the structures of the corresponding immunity proteins CdiIs are diverse.

In protein translation, GTP-bound Tu (Tu:GTP) delivers aminoacyl-tRNAs (aa-tRNAs) to the ribosome A-site (18). When a codon–anticodon match occurs on the A-site, GTP is hydrolyzed to GDP by Tu, and GDP-bound Tu (Tu:GDP) is released from the ribosome (19,20). Ts binds Tu:GDP, displaces GDP, and recycles Tu, thus acting as a guanine nucleotide exchange factor (GEF) for Tu (21,22). Translation factors have functions beyond protein synthesis, e.g. in the replication of Qβ phage RNA by Qβ replicase, which consists of a phage-encoded RNA-dependent RNA polymerase (RdRp: β-subunit) along with host-derived Tu, Ts, and ribosomal protein S1 (23,24). In Qβ replicase, Tu and Ts function as chaperones (25) and RNA elongation cofactors that facilitate the separation of the template and growing RNAs during replication (26). The chaperone function of Tu was described (27–29), but its detailed mode of action has remained enigmatic.

When Tu, Ts and GTP were first described to be required for cleavage of the 3′-acceptor regions of specific tRNAs by CdiA–CT^EC869^, it was proposed that CdiA–CT^EC869^ recognizes an aa-tRNA complexed with Tu:GTP (15). The same scenario was proposed for CdiA–CT^NC101^, which also requires Tu, Ts, and GTP for specific tRNA cleavage (14). However, in these studies, the authors only tested cleavage of uncharged tRNAs, not aa-tRNAs, and neither the effect of the aminoacyl status of tRNA nor the kinetics of tRNA cleavage in the presence of translation factors has been well examined. A subsequent study showed that CdiA–CT^Kp342^ cleaves uncharged tRNA^Ile^, rather than Ile-tRNA^Ile^, in the presence of translation factors *in vitro*, and that the closely related *E. coli* 3006 CdiA–CT, CdiA–CT^EC3006^, whose tRNase activity does not require Tu and Ts, cleaves uncharged tRNA^Ile^ between positions 70 and 71, but not Ile-tRNA^Ile^ (16). Therefore, unanswered questions remain about the mechanism underlying substrate tRNA recognition by these CdiA–CTs targeting the 3′-acceptor region of tRNAs, their specificity, and the functions of translation factors in the enhancement of tRNA cleavage by the above-mentioned CdiA–CTs (17).

In this study, we analyzed the CdiA toxin from enterohemorrhagic *E. coli* EC869, CdiA–CT^EC869^ (15). Our biochemical and structural analyses collectively suggest that tRNA cleavage by CdiA–CT^EC869^ in the presence of Tu, Ts, and GTP proceeds via the CdiA–CT^EC869^:Tu:GTP:Ts:tRNA complex. CdiA–CT^EC869^ is recruited to Tu:GTP:Ts and forms CdiA–CT^EC869^:Tu:GTP:Ts complex, which recognizes tRNA and aa-tRNA. Tu:GTP:Ts in the complex serves as a reaction scaffold that facilitates tRNA cleavage by CdiA–CT^EC869^ by increasing the affinity of CdiA–CT^EC869^ for the tRNA substrate and inducing a structural change of the 3′-portion of the tRNA with assistance from Ts in the complex.

## MATERIALS AND METHODS

### Plasmid construction

Synthetic DNA containing the *cdiA-CT/cdiI* module from enterohemorrhagic *E. coli* EC869 was purchased from Eurofins, Japan. The nucleotide sequence of the *cdiA-CT/cdiI*^EC869^ module is provided in Supplementary Table S1. For overexpression of the CdiA–CT/CdiI^EC869^ complex in *E. coli*, the *cdiA-CT/cdiI*^EC869^ module was PCR amplified and cloned between the NdeI and XhoI sites of pET15L, yielding pET15L-CdiA–CT/CdiI^EC869^. To generate plasmids for expression of the inactive CdiA–CT (H281A or ΔC5) mutant proteins, the mutations were introduced by PCR into the CdiA–CT^EC869^ coding region, and the resultant PCR fragments were cloned into the NdeI and XhoI sites of pET15L, yielding the plasmids pET15L-CdiA–CT^EC869^_H281A and pET15L-CdiA–CT^EC869^_ΔC5. For expression of CdiI^EC869^, the *CdiI* gene was also PCR amplified and cloned between the NdeI and XhoI sites of pET15L and pET15sumo, yielding plasmids pET15L-CdiI^EC869^ and pET15sumo-CdiI^EC869^, respectively. pET15sumo was designed to express proteins fused with an N-terminal Sumo-tag carrying the N-terminal hexahistidine (His_6_) sequence.

The DNA fragment containing the *cdiA-CT/cdiI*^EC869^ module was cloned between the NdeI and HindIII sites of pBAD33 (ATCC87402), yielding pBAD33_CdiAI^EC869^. pBAD33_CdiAI^EC869^ variants with a mutation in the CdiA–CT coding region were prepared by PCR. pBAD33_CdiA^EC869^_H281A and pBAD33_CdiA^EC869^_ΔC5 were constructed by PCR amplification of the CdiA coding regions of pET15L-CdiA–CT^EC869^_H281A and pET15L-CdiA–CT^EC869^_ΔC5, which were then cloned between the NdeI and HindIII sites of pBAD33. Expression plasmids for *E. coli* Tu and Ts were described previously (25). A gene for single chain Tu-Ts was designed as previously described (30). A DNA fragment encoding Tu, Ts or Tu-Ts was cloned into the NdeI and XhoI sites of pET15sumo, yielding plasmids pET15sumo-EF-Tu, pET15sumo-EF-Ts and pET15sumo-EF-Tu-Ts, respectively. The *E. coli* glutaminyl-tRNA synthase gene was PCR amplified from *E. coli* genomic DNA and cloned between the NdeI and XhoI sites of pET22b, yielding pET22EcGlnRS.

A synthetic DNA containing the *E. coli* tRNA^Gln^ gene harboring the U1G mutation, driven by the T7 RNA promoter, was purchased from Eurofins and cloned between the EcoRI and HindIII sites of pUC19, yielding pT7EctRNAGlnG1A72. Other plasmids for *in vitro* transcription of tRNA^Gln^ variants, precursor tRNA^Gln^ variants, and tRNA^Trp^ were purchased from Eurofins, and the DNA sequences used for *in vitro* transcription are listed in Supplementary Table S1. The oligonucleotide primers used for the PCR cloning and mutations are listed in Supplementary Table S2.

### Protein purification

For expression of CdiA–CT/CdiI^EC869^, *E. coli* Rosetta1 cells (Novagen-Merck Millipore) were transformed with pET15L-CdiA–CT/CdiI^EC869^ and cultured until the OD_600_ reached ∼0.6. Protein expression was induced by addition of 1 mM of isopropyl-β-d-thiogalactopyranoside (IPTG), and culture was continued for 2 h at 37°C. For expression of CdiI^EC869^, CdiA–CT^EC869^_H281A, and CdiA–CT^EC869^_ΔC5, cells were transformed with pET15L-CdiI^EC869^, pET15L-CdiA–CT^EC869^_H281A and pET15L-CdiA–CT^EC869^_ΔC5. For expression of Tu, Ts and their variants, E. coli BL21(DE3) was transformed with pET22EF-Tu, pET22-Ts and their variants, respectively, and protein expression was induced by addition of 0.1 mM IPTG followed by culture for 20 h at 20°C. The harvested cells were sonicated in Ni-Buffer [50 mM Tris–Cl, pH 7.0, 500 mM NaCl, 10% (v/v) glycerol, 20 mM imidazole, 5 mM β-mercaptoethanol] supplemented with 0.1 mM PMSF (phenylmethylsulfonyl fluoride) and 50 μg/ml lysozyme. The lysates were centrifuged at 30 000 × g and 4°C for 1 h, and the supernatants were applied to a Ni-NTA column (QIAGEN, Japan). The column was washed with Ni-Buffer and the proteins were eluted with Ni-Buffer supplemented with 400 mM imidazole. The proteins were then applied to a Hi-Trap Heparin or Hi-Trap Q column (GE Healthcare, Japan). Finally, the proteins were purified on a Hi-Load 16/60 Superdex 200 column (GE Healthcare, Japan), equilibrated with buffer containing 25 mM Tris–Cl, pH 7.0, 150 mM NaCl, and 10 mM β-mercaptoethanol, and concentrated. For the purification of proteins without the His_6_-tag, SUMO (small ubiquitin-related modifier)-tagged proteins carrying His_6_ at their N-termini were expressed and purified by Ni-NTA chromatography as described above. The SUMO tags were removed from the proteins by digestion at 4°C overnight with Ulp1 (Ubl-specific protease 1). Subsequently, the SUMO-tag–free proteins were purified by applying to Ni-NTA column chromatography, and the flow-through fractions were collected. The proteins were further purified as described above.

For isolation of CdiA–CT^EC869^, the purified CdiA–CT/CdiI^EC869^ protein complex was denatured on a Ni-NTA column equilibrated with buffer containing 8 M urea, 500 mM NaCl, and 25 mM Tris–Cl, pH 8.0 (31–33). Denatured CdiI^EC869^ protein was removed from the column by washing the column with the denaturing buffer. The denatured CdiA–CT^EC869^ protein was refolded with a stepwise gradient of urea (6, 4, 2, 0.5 and 0 M) on the column, and the renatured CdiA–CT^EC869^ protein was eluted from the column with Ni-Buffer supplemented with 300 mM imidazole. The purified CdiA–CT^EC869^ protein was then applied to a Hi-Trap Heparin column (GE Healthcare, Japan) and further purified on a Hi-Load 16/60 Superdex 200 column (GE Healthcare, Japan), equilibrated with 25 mM Tris–Cl, pH 7.0, 150 mM NaCl, and 10 mM β-mercaptoethanol. To prepare nucleotide-free wild-type Tu, His84Ala mutant Tu and sg–Tu–Ts, the purified proteins were incubated for 20 min at 37°C in Mg^2+^-free buffer containing 25 mM Tris–Cl, pH 6.8, 150 mM NaCl, 10 mM β-mercaptoethanol, and 10 mM EDTA, as described (34). The samples were then purified by gel filtration on a Hi-Load 16/60 Superdex 200 column (GE Healthcare, Japan) in the same buffer without Mg^2+^.

### tRNA preparation

For preparation of tRNA^Gln^ transcript variants starting with A1, C1 or U1, the corresponding precursor tRNAs (pre-tRNAs) with 5′-leader sequences were synthesized by T7 RNA polymerase using plasmid linearized by *Fok*I digestion. The 5′-leader RNA was processed *in vitro* by RNase P, which consists of the M1 RNA and C5 protein (33). The pre-tRNA transcript was processed in a mixture containing 100 nM M1 RNA, 100 nM C5 protein, 50 mM Tris–Cl, pH 7.4, 10 mM MgCl_2_ and 100 mM NH_4_Cl at 37°C overnight, followed by phenol extraction and isopropanol precipitation. The processed mature tRNAs were dissolved in a buffer containing 20 mM Tris–Cl, pH 7.4 and 200 mM NaCl, applied to a Resource Q column (GE Healthcare, Japan), and separated by a linear NaCl gradient (0.2–1.0 M) in the buffer. tRNAs were ethanol-precipitated, rinsed, and dried. The tRNA_f_^Met^ and tRNA^Trp^ transcripts were also prepared as described above (33). tRNA^Gln^ G1 variants were synthesized using T7 RNA polymerase and purified as described above.

### *In vitro* tRNA cleavage assay

The standard CdiA–CT^EC869^ tRNase activity assay was conducted at 37°C in reaction buffer containing 20 mM Tris–Cl, pH 7.4, 150 mM NaCl, 5 mM MgCl_2_, 10 mM β-mercaptoethanol, 100 μg/ml bovine serum albumin (BSA), 1 μM tRNA^Gln^ transcript (or its variant), 0.1 μM CdiA–CT^EC869^, 0.1 μM Tu, 0.1 μM Ts and 1 mM GTP. Reactions were quenched by addition of an equal volume of formamide gel-loading buffer [95% (v/v) formamide, 0.02% (w/v) SDS, 0.02% (w/v) bromophenol blue, 0.01% (w/v) xylene cyanol] at the indicated time points, and the tRNAs were separated by 10% (w/v) polyacrylamide gel electrophoresis (PAGE) under denaturing conditions. The gels were stained with ethidium bromide, and tRNA band intensities were quantified by the Image Lab software (Bio-Rad, version 3.0). The specific conditions for each assay of tRNA cleavage by CdiA–CT^EC869^ are clearly described in either the text or figure legends.

### Whole-mass analysis of tRNAs

tRNA^Gln^, tRNA^Trp^, and tRNA_f_^Met^ transcripts were cleaved by CdiA–CT in the presence of translation factors and GTP, and the cleaved products were subjected to 10% (w/v) PAGE under denaturing conditions. A total of 10 pmol of each tRNA was subjected to reverse phase (ODS) capillary liquid chromatography (LC) coupled to nano electrospray (ESI)/mass spectrometry (MS) on a linear ion trap-Orbitrap hybrid mass spectrometer (LTQ Orbitrap XL; Thermo Fisher Scientific) as described previously (35,36). RNAs were scanned in a negative polarity mode and detected by a linear ion trap device. The scanning range was set to 600–2000. A multiply-charged ESI spectrum was deconvoluted by ProMass HR for Xcalibur (Novatia, LLC) to calculate the average molecular mass.

### *In vivo* toxicity assay

*E. coli* strain MG1655 (NIG, Japan; ME7986) was transformed with pBAD33_CdiA–CT^EC869^ its variants, inoculated into LB containing 50 μg/ml chloramphenicol and 1% (w/v) glucose, and cultured at 37°C. The toxicities of CdiA–CT^EC869^ and its variants were evaluated by spot assay. Specifically, overnight LB cultures were serially diluted, 3-μl aliquots of the dilutions were spotted on LB agar plates containing 50 μg/ml chloramphenicol supplemented with 1% (w/v) arabinose or 1% (w/v) glucose, and the plates were incubated overnight at 37°C.

### Preparation of aa-tRNAs and LC/MS spectrometry

*E. coli* strain MG1655 was transformed with pBAD33_CdiA–CT^EC869^ or control pBAD33, inoculated in LB containing 50 μg/ml chloramphenicol and 1% (w/v) glucose, and cultured overnight at 37°C. The overnight cultures were diluted to an OD_660_ of 0.02 in LB (4 ml) containing 50 μg/ml chloramphenicol and cultured at 37°C until the OD_660_ reached 0.3, and then 0.02% (w/v) arabinose was added. After a 10-min incubation, the cells were harvested and suspended in buffer containing 50 mM NaOAc, pH 5.0, 0.5 mM EDTA, and 0.2 M NaCl. RNA was extracted under acidic conditions using phenol saturated with 300 mM NaOAc, pH 5.2, followed by isopropyl alcohol precipitation. The precipitated RNA was dissolved in 100 μl of 300 mM NaOAc, pH 5.2. aa-tRNAs were chemically acetylated by addition of acetic anhydride (32,33,37) and then ethanol-precipitated and rinsed with 70% cold ethanol. The RNA was dissolved in cold buffer containing 50 mM NaOAc, pH 5.0, 0.5 mM EDTA, and 0.2 M NaCl, and loaded onto a 100-μl Q-Sepharose F.F. (GE Healthcare, Japan) column. The resin was washed with buffer containing 50 mM NaOAc, pH 5.0, 0.5 mM EDTA and 0.3 M NaCl. Finally, the tRNA fraction was eluted with buffer containing 50 mM NaOAc, pH 5.0, 0.5 mM EDTA and 0.6 M NaCl, ethanol-precipitated, and rinsed with 70% (v/v) ethanol. The acetylated aa-tRNAs were digested with RNase One Ribonuclease (Promega, Japan) at 37°C for 60 min in a 25-μl reaction mixture containing 25 mM NH_4_Oac and 2.5 units enzyme. The digests were subjected to an LC/MS analysis using a Q Exactive Hybrid Quadrupole–Orbitrap Mass Spectrometer (Thermo Fisher Scientific) equipped with a Dionex UltiMate 3000 LC System (Thermo Fisher Scientific) and an InertSustain C18 column (5 μm, 2.1 × 250 mm, GL Sciences), as described (32,33,38).

### Gel-shift assay

tRNA^Gln^ was uniformly ^32^P-labeled with α-^32^P UTP (PerkinElmer, Japan, 3,000 Ci/mmol) using an in vitro transcription kit (Promega, Japan) and then gel-purified. The ^32^P-labeled tRNA^Gln^ (10 000 cpm) was incubated in 10 μl of a solution containing 50 mM Tris–Cl, pH 7.4, 10 mM MgCl_2_, 50 mM NaCl, 10 mM β-mercaptoethanol, 10% (v/v) glycerol and various amounts of CdiA–CT^EC869^_H281A, Tu, or single-chain Tu-Ts (sg–Tu–Ts), at 37°C for 10 min. The solutions were then cooled on ice. The solutions were separated by 6% (w/v) native acrylamide gel electrophoresis (1 × TBE) at room temperature (∼25°C), as described (39). ^32^P-labeled bands were quantified using a BAS-5000 imager (FujiFilm, Japan).

### Northern blotting

*E. coli* tRNA mixtures used for the *in vitro* cleavage assay by CdiA–CT^EC869^ were prepared from JM109tr as described (31). The RNA fraction was deacylated by incubation in 1.8 M Tris–Cl, pH 8.0, for 2 h at 37°C and separated by Hi-Load 16/10 Q-Sepharose HP chromatography, and then the tRNA fractions were pooled and ethanol-precipitated. RNAs with or without CdiA–CT^EC869^ treatment were separated on a 10% (w/v) polyacrylamide gel containing 7 M urea and then transferred to a Hybond-N + membrane (GE Healthcare, Japan) by a Trans-Blot SD semi-dry cell (Bio-Rad, Japan). Hybridization was carried out overnight at 60°C in PerfectHyb Hybridization Solution (Toyobo, Japan). The membrane was washed three times with buffer containing 2 × standard saline citrate (SSC) and 0.1% SDS for 5 min and then washed twice with buffer containing 0.1 × SSC and 0.1% (w/v) SDS for 10 min at 60°C. The oligonucleotide sequences used as specific probes for tRNAs are listed in Supplementary Table S2.

### Pull-down assay

Interactions between CdiA–CT^EC869^ and translation factors in the presence of tRNA/aa-tRNA were analyzed by a pull-down assay. Nucleotide-free Tu was preincubated with a 10-fold excess of GTP for 15 min at 37°C in buffer A (50 mM Tris–Cl, pH 7.0, 50 mM NH_4_Cl, 10 mM MgCl_2_ and 5 mM β-mercaptoethanol) as described previously (34). To prepare Gln-tRNA^Gln^, tRNA^Gln^ was aminoacylated by GlnRS in buffer containing 30 mM Tris–HCl, pH 7.4, 15 mM MgCl_2_, 25 mM KCl, 5 mM DTT, 4 mM ATP and 2 mM glutamine as described previously (40). After incubation for 1 h at 37°C, RNA was extracted under cold acidic conditions using phenol saturated with 300 mM NaOAc, pH 5.2, followed by isopropyl alcohol precipitation. The precipitated RNA was dissolved in 300 mM NaOAc, pH 5.2, and purified using a NAP5 column (Amersham Biosciences, Japan), followed by isopropyl alcohol precipitation and a rinse with cold 70% (v/v) ethanol. The precipitated RNA was dissolved in 20 mM NaOAc, pH 5.2 and used in the pull-down assay.

Hexahistidine-tagged catalytic dead CdiA–CT^EC869^_H281A (5 μM) was mixed with an equimolar amount of translation factors in the presence of an equimolar amount of tRNA/aa-tRNA in buffer A containing 1 mM GTP. The solution (200 μl volume) was incubated for 5 min at 37°C and placed on ice. To this solution, 150 μl of Ni-NTA resin equilibrated with wash buffer (25 mM Tris–Cl, pH 7.0, 100 mM NaCl, 5 mM MgCl_2_, 5 mM NH_4_Cl, 5 mM β-mercaptoethanol, 10 mM imidazole, and 1 mM GTP) was added, and the mixture was incubated at 4°C for 60 min. The mixture was loaded onto a poly-prep chromatography column (Bio-Rad, Japan). The Ni-NTA resin was washed with 10 column volumes of wash buffer and eluted with the same buffer containing 300 mM imidazole. The fractions were separated by 12% (w/v) SDS PAGE to visualize proteins or 10% (w/v) polyacrylamide gel containing 7 M urea to visualize tRNA.

### Crystallization and structural determination of Tu:CdiA–CT:CdiI complex

For crystallization of the Tu:CdiA–CT:CdiI^EC869^ ternary complex, 36 μM CdiA–CT:CdiI complex was mixed with an equimolar amount of Tu and incubated at 4°C for 30 min. A 1-μl aliquot of the protein mixture solution was mixed with 1.0 μl of reservoir solution containing 100 mM HEPES–KOH, pH 7.5, 2% (w/v) Tacsimate, pH 7.0, and 22% (w/v) PEG3350, and the crystals were generated by the hanging-drop vapor diffusion method at 20°C. The crystals were flash-cooled in 1.1 × concentrated reservoir solution, supplemented with 20% (v/v) ethylene glycol as a cryoprotectant. Data sets were collected on beamline 17A at the Photon Factory at KEK, Japan. The data were indexed, integrated, and scaled with XDS (41). The crystal belongs to space group *C*121 with one CdiA–CT:CdiI^EC869^ :Tu complex in the asymmetric unit cell. The initial phase was determined by the molecular replacement method, using the structure of *E. coli* Tu (PDB ID: 4PC3) as the search model. Although the electron densities corresponding to Tu were visible, the electron density corresponding to CdiA–CT:CdiI was not. We modeled CdiA–CT and CdiI structures using AlphaFold 2 (42). Molecular replacement using the modeled CdiA–CT and CdiI structures as search models improved the electron densities corresponding to CdiA–CT:CdiI. The structure was refined with phenix.refine (43), and manually modified with Coot (44). Finally, the structure was model-built and refined to an *R* factor of 23.7% (*R*_free_ = 27.8%) at 3.41 Å resolution. The modeled CdiA–CT contains residues 175–285 (numbered from Val1 of the VENN peptide motif), and the modeled CdiI^EC869^ contains residues 3–179. The modeled Tu contains residues 10–393. The details of the crystallographic data collection and refinement statistics are provided in Table 1.

**Table 1.** Data collection and refinement statistics

	Tu:CdiA–CT:CdiI
**Data collection**	
Space group	*C* 1 2 1
Cell dimensions	
*a*, *b*, *c* (Å)	101.93, 102.76, 88.14
α, β, γ (°)	90, 111.863, 90
Wavelength (Å)	0.98000
Resolution (Å)^a^	49.76–3.40 (3.52–3.40)
*R*_sym_^a^	0.308 (2.134)
*I* / σ*I*^a^	7.1 (1.0)
*CC*_1/2_a	0.994 (0.386)
Completeness (%)^a^	98.4 (89.0)
Redundancy^a^	7.5 (6.7)
**Refinement**	
Resolution (Å)	35.6–3.413
No. reflections	86153
*R*_work_ / *R*_free_ (%)	23.68 / 27.79
No. atoms	
Protein	5190
Ligand	-
Water	-
*B*-factors (Å^2^)	
Protein	111.13
Ligand	-
Water	-
R.m.s. deviations	
Bond lengths (Å)	0.004
Bond angles (°)	1.04

^a^Values in parentheses are for the highest resolution shell.

## RESULTS

### Translation factors promote tRNA cleavage by CdiA–CT^EC869^

The specific tRNA cleavage reaction catalyzed by the C-terminal domain of CdiA from enterohemorrhagic *E. coli* EC869 (CdiA–CT^EC869^; hereafter simply CdiA–CT) requires translation elongation factors Tu and Ts as well as GTP, although GTP hydrolysis is not required (15).

**Figure 1.**
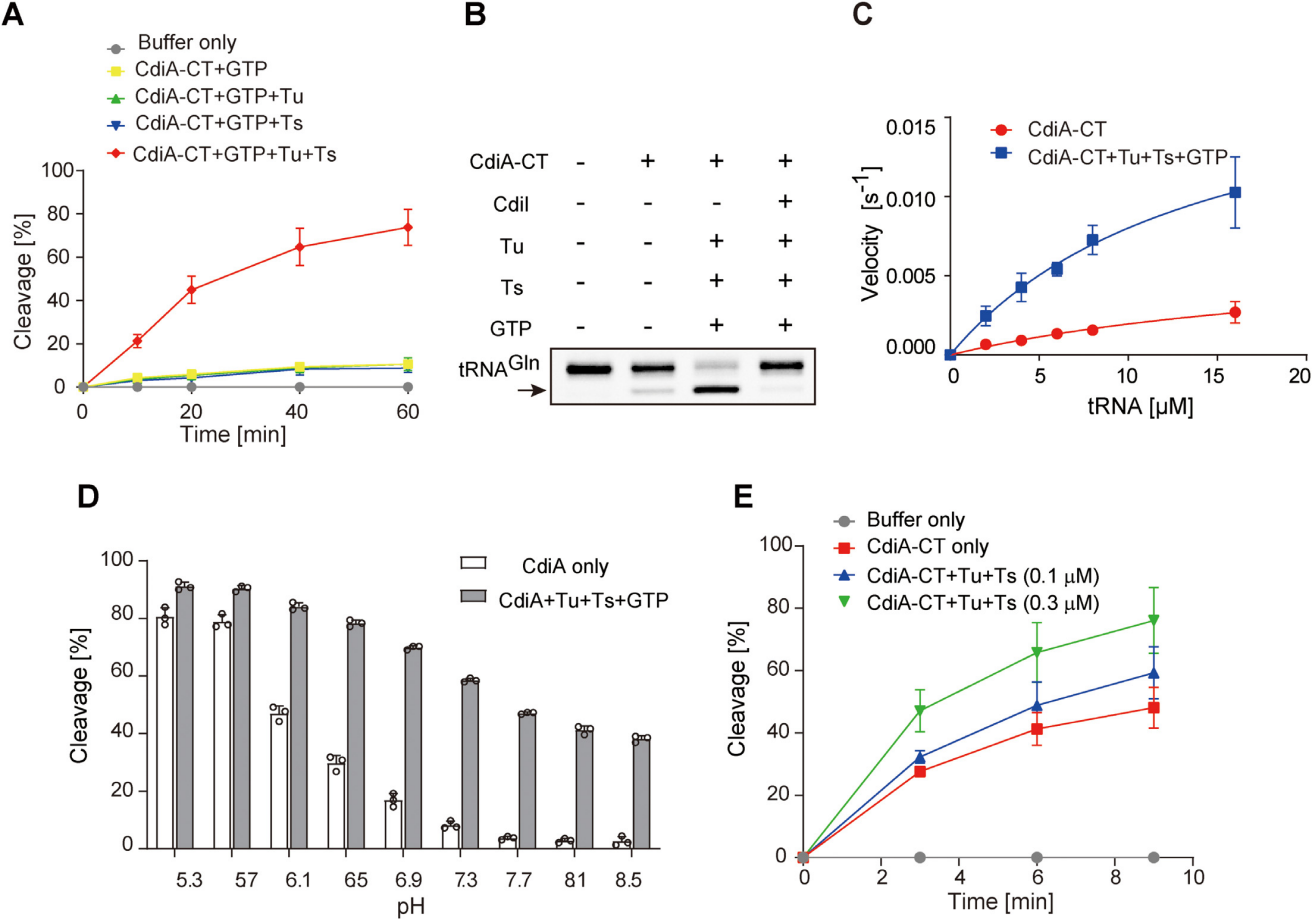
tRNA cleavage by CdiA–CT^EC869^ in the presence of translation factors and GTP. (**A**) tRNA^Gln^ cleavage by CdiA–CT is dependent on Tu, Ts and GTP under neutral pH conditions (pH = 7.4). tRNA^Gln^ (1 μM) was incubated at 37°C with 0.1 μM CdiA–CT in the presence of Tu (0.1 μM), Ts (0.1 μM), and GTP (1 mM), and the tRNA was separated by 10% (w/v) PAGE under denaturing conditions. The fraction of cleaved tRNA was quantified at the indicated time points. (**B**) tRNA^Gln^ cleavage is blocked by CdiI^EC869^, an immunity protein against CdiA–CT^EC869^. CdiA–CT was preincubated with an equimolar amount of CdiI^EC869^ before the reaction and then subjected to the cleavage reaction for 60 min at 37°C. (**C**) Steady-state kinetics of tRNA^Gln^ cleavage by CdiA–CT^EC869^ in the presence or absence of Tu, Ts, and GTP. The initial velocities of tRNA^Gln^ cleavage by CdiA–CT^EC869^ were measured at various concentrations of tRNA^Gln^ (2–16 μM). Tu (0.1 μM), Ts (0.1 μM) and GTP (1 mM) were added to CdiA–CT^EC869^ (0.1 μM) to calculate the initial velocities of tRNA cleavage by CdiA–CT^EC869^ in the presence of translation factors at pH 7.4. (**D**) CdiA–CT activity is pH-dependent and elevated under lower pH conditions. The cleavage of tRNA^Gln^ by CdiA–CT alone (open bars) and by CdiA–CT^EC869^ in the presence of Tu, Ts, and GTP (hatched bars) is shown under various pH conditions. The tRNA^Gln^ transcript (1.0 μM) was incubated for 10 min at 37°C with 0.2 μM of CdiA–CT in the presence or absence of Tu, Ts, and GTP. The Y-axis in the graph represents cleavage (%) at 10 min. For pH ranges from 5.3 to 6.9 and 7.3 to 8.5, 20 mM MES and 20 mM Tris–Cl buffers were used, respectively. The tRNA was separated by 10% (w/v) PAGE under denaturing conditions, and the fraction of the cleaved tRNA was quantified. (**E**) tRNA^Gln^ cleavage by CdiA–CT under acidic conditions (pH 5.3) is still, but to a lesser extent, dependent on the presence of Tu, Ts, and GTP. tRNA^Gln^ (1.0 μM) was incubated with 0.05 μM CdiA–CT^EC869^ in the presence of increasing amounts of Tu, Ts (0, 0.1 and 0.3 μM each), and GTP (1 mM) at 37°C. The bars in the graphs are SDs of more than three independent experiments, and the data are presented as mean values ± SD.

To confirm the requirement of translation factors and GTP for tRNA cleavage by CdiA–CT, we prepared highly purified recombinant Tu, Ts and CdiA–CT by several chromatography steps (Supplementary Figure S1). Because Tu and Ts form Tu:Ts heterodimer (45), Tu (or Ts) was purified with great care to avoid the contamination of Ts (or Tu) in the preparations. We then tested the tRNA cleavage by CdiA–CT in the presence of translation factors using the tRNA^Gln^ transcript as the substrate *in vitro*. Under standard test conditions (pH 7.4), the tRNA cleavage level was increased in the presence of Tu, Ts, and GTP (Figure 1A). tRNA cleavage was suppressed by the prior incubation of CdiA–CT with its immunity protein, CdiI^EC869^, confirming that the observed tRNA cleavage was caused by CdiA–CT (Figure 1B).

In these analyses, even in the absence of translation factors and GTP, CdiA–CT could cleave tRNA^Gln^ to some extent *in vitro* (Figure 1A, B). We analyzed the steady-state kinetics of tRNA cleavage by CdiA–CT with or without Tu, Ts, and GTP. In the absence of Tu, Ts, and GTP, the apparent *K*_m_ value of tRNA^Gln^ was 24.5 ± 10.2 μM, and *k*_cat_ was 0.0068 ± 0.002 s^–1^. On the other hand, in the presence of Tu, Ts and GTP, the apparent *K*_m_ value of tRNA^Gln^ was 14.4 ± 4.1 μM, and *k*_cat_ was 0.020 ± 0.003 s^–1^ (Figure 1C). Although it is unclear what the apparent *K*_m_ value of tRNA^Gln^ in the presence of Tu:GTP:Ts represents in this analysis, these results imply that a putative Tu:GTP:Ts complex increases the overall catalytic efficiency of processing catalyzed by CdiA–CT.

**Figure 2.**
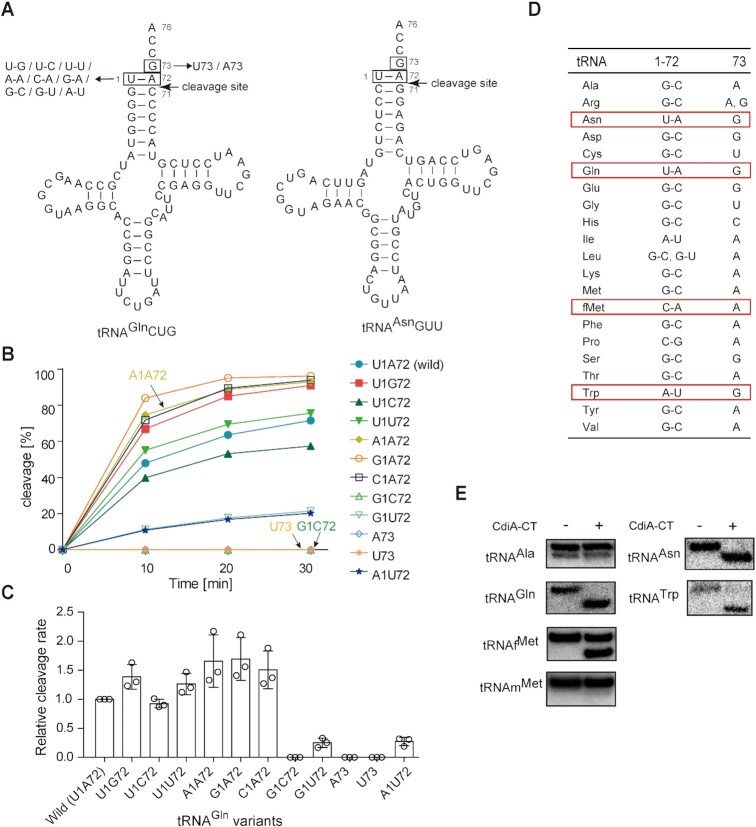
Recognition elements in tRNA for cleavage by CdiA–CT^EC869^. (**A**) Nucleotide sequence and secondary cloverleaf structure of *E. coli* tRNA^Gln^ and tRNA^Asn^. The mutations introduced into the tRNA^Gln^ transcript are depicted on the left. (**B**) Time course of the cleavage of tRNA^Gln^ variants in (A) by CdiA–CT^EC869^ under neutral pH conditions (pH 7.4). tRNA^Gln^ transcript variants (1.0 μM) were incubated at 37°C with 0.2 μM CdiA–CT^EC869^ in the presence of Tu (0.2 μM), Ts (0.2 μM) and GTP (1 mM). The fraction of cleaved tRNA was quantified at the indicated time points as in (A). (**C**) Relative cleavage of tRNA^Gln^ variants by CdiA–CT in the presence of Tu, Ts, and GTP as in (B), at 37°C. Cleavage level of wild-type tRNA^Gln^ at 10 min was taken as 1.0. The error bars in the graphs represent SDs of at least three independent experiments, and the data are presented as mean values ± SD. (**D**) Comparison of nucleotide compositions at positions 1, 72, and 73 of 20 kinds of tRNAs in *E. coli*. (**E**) Cleavage of total tRNAs prepared from *E. coli* by CdiA–CT *in vitro*. Total tRNA mixtures (0.5 μg) were incubated at 37°C for 60 min with 0.5 μM CdiA–CT^EC869^ (20 μl reaction solution) in the presence of Tu (0.5 μM), Ts (0.5 μM) and GTP (1 mM). Cleavage of specific tRNAs was detected by northern blotting using DNA oligonucleotides specific for the corresponding tRNAs (tRNA^Ala^, tRNA^Asn^, tRNA^Gln^, tRNA^Trp^, tRNA_f_^Met^ and tRNA_m_^Met^).

As described above, CdiA–CT alone could cleave tRNA. Thus, the reaction conditions used for the measurement of tRNA cleavage by CdiA–CT were evaluated. Under lower pH conditions (i.e. below 6.9) in the presence of Tu, Ts and GTP, CdiA–CT cleaved tRNA more efficiently than under neutral or basic pH conditions (Figure 1D). Furthermore, even in the absence of Tu, Ts and GTP, under more acidic conditions (pH 5.7 and 5.3), CdiA–CT alone could cleave tRNA to almost the same extent as it could in the presence of Tu, Ts, and GTP (Figure 1D). To examine the promotion of tRNA cleavage by CdiA–CT in the presence of translation factors under acidic conditions, we evaluated the effects of Tu, Ts, and GTP on tRNA cleavage by CdiA–CT. The tRNA cleavage level by CdiA–CT at pH 5.3 was still elevated, but to a lesser extent, when Tu, Ts and GTP were present (Figure 1E). Together with the analysis of the substrate specificity of CdiA–CT using various tRNAs, described below (Figure 2), these observations suggest that the substrate specificity is governed by CdiA–CT itself and that the translation factors and GTP efficiently enhance tRNA cleavage by CdiA–CT at neutral pH. The mechanism underlying the increase in enzymatic activity of CdiA–CT under acidic conditions remains elusive. Under acidic conditions, the structure of the 3′-acceptor region of tRNA might be altered, allowing CdiA–CT to more efficiently cleave tRNAs as described below. Alternatively, the catalytic process might be accelerated by protonation of key catalytic residues, although the precise mechanism for such an effect remains unknown.

### Substrate tRNA recognition by CdiA–CT^EC869^*in vitro*

CdiA–CT reportedly cleaves tRNA^Gln^ and tRNA^Asn^ between positions 71 and 72 (15). To clarify CdiA–CT recognition sites in the substrate tRNAs, we prepared tRNA^Gln^ transcript variants (Figure 2A) based on comparison with the tRNA^Asn^ sequence, and then their cleavage by CdiA–CT was examined *in vitro*. Under standard conditions (pH 7.4), in the presence of Tu, Ts and GTP, mutant tRNA^Gln^ in which the discriminator G73 was replaced with A73 or U73 could not be efficiently cleaved by CdiA–CT (Figure 2B, C), suggesting that the discriminator at position 73 is a strong determinant of CdiA–CT recognition of substrate tRNAs. A tRNA^Gln^ variant with a G1–C72 base pair at the top of the acceptor stem was also inefficiently cleaved. On the other hand, tRNA^Gln^ variants with mis-pairings (A1–A72, G1–A72, U1–U72, U1–G72, U1–C72 or C1–A72) at the top of the acceptor stem were cleaved as efficiently as wild-type tRNA^Gln^ (Figure 2C). On the other hand, tRNA^Gln^ variants with the G1–U72 or A1–U72 mutation were less efficiently cleaved. Collectively, these observations suggest that the presence of the G73 discriminator, a mismatch, a weaker base pair at the top of the acceptor helix, and pyrimidine and purine bases at positions 1 and 72, respectively, are important for the cleavage of specific tRNAs by CdiA–CT. We also examined cleavage of tRNA^Gln^ variants by CdiA–CT alone under lower pH conditions (pH 5.7). The substrate specificity of CdiA–CT (Supplementary Figure S2B) was essentially identical to that observed under standard conditions (pH 7.4) in the presence of Tu, Ts, and GTP (Figure 2C). These results support the idea that CdiA–CT itself has an intrinsic ability to recognize and select specific tRNA substrates.

Next, we compared the nucleotide compositions at positions 1, 72 and 73 of all tRNAs from *E. coli* (Figure 2D, Supplementary Figure S3). Among the 20 kinds of isoaccepting tRNAs, tRNA^Gln^ and tRNA^Asn^ have G73 and the weak U1–A72 base pair. Thus, both tRNA^Gln^ and tRNA^Asn^ can be efficiently cleaved by CdiA–CT, as described above. tRNA^Trp^ has a weak A1–U72 base pair and G73, and initiator tRNA_f_^Met^ has a C1–A72 mismatch but A73. We investigated whether tRNA^Trp^ and tRNA_f_^Met^ (Supplementary Figure S4A) could be cleaved by CdiA–CT. Total *E. coli* tRNA was treated with CdiA–CT under the standard conditions (pH 7.4) in the presence of Tu, Ts, and GTP, and tRNA cleavage was detected by northern hybridization. The results revealed that in addition to tRNA^Gln^ and tRNA^Asn^, tRNA^Trp^ and tRNA_f_^Met^ were also cleaved *in vitro* (Figure 2E). Analysis of the cleavage sites in tRNA_f_^Met^ and tRNA^Trp^ revealed that both tRNA_f_^Met^ and tRNA^Trp^ were cleaved between positions 71 and 72 as tRNA^Gln^ yielding terminal 2′-3′-cyclic phosphate ends (Supplementary Figure S4B–D). While tRNA^Trp^ was cleaved almost as efficiently as tRNA^Gln^, tRNA_f_^Met^ was less efficiently cleaved than tRNA^Gln^ by CdiA–CT in the presence of translation factors *in vitro* (Figure 2E, Supplementary Figure S4E).

**Figure 3.**
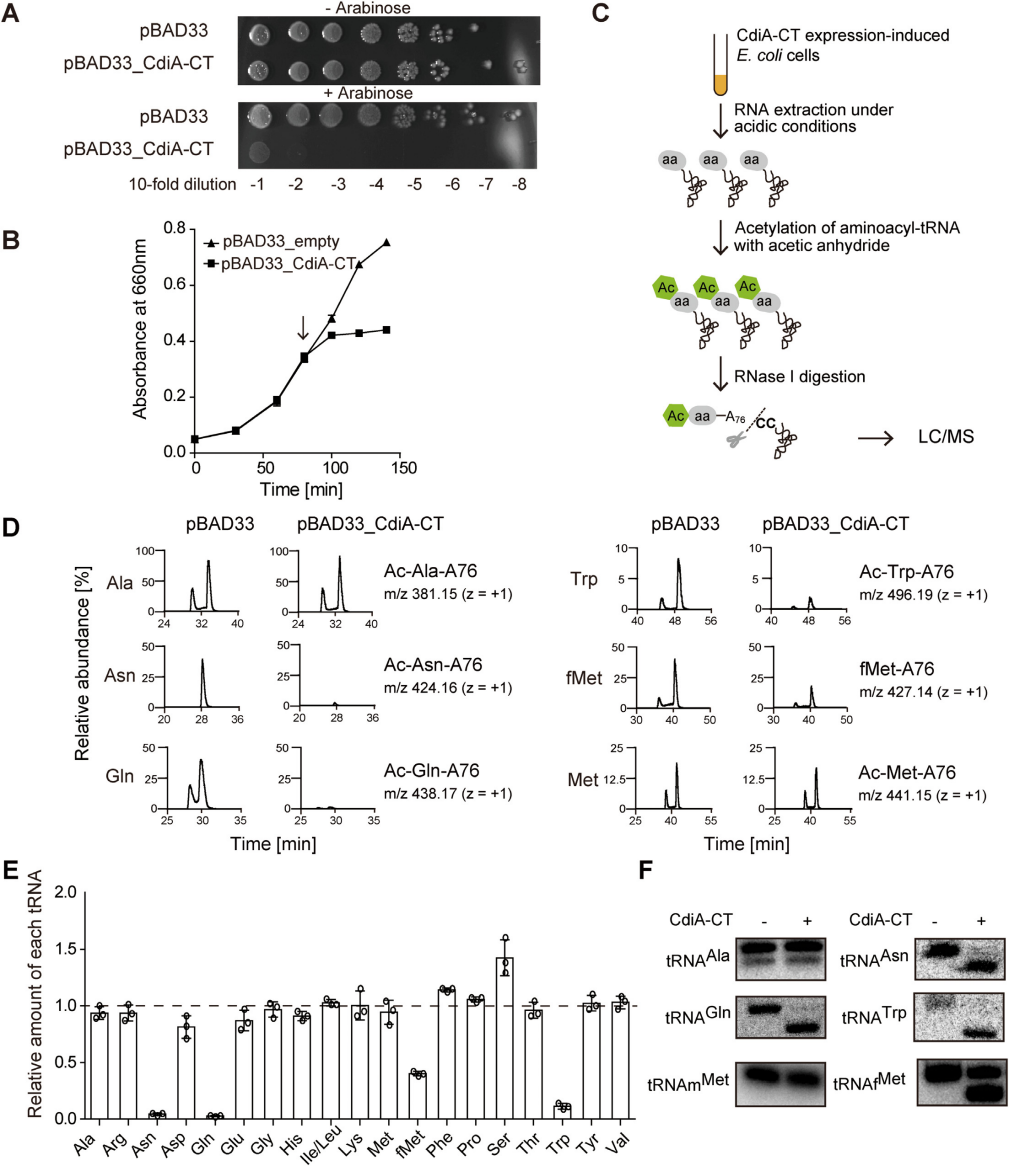
Changes in aa-tRNA levels upon CdiA–CT^EC869^ induction *in vivo*. (**A)** Growth inhibition by CdiA–CT^EC869^ induction. Overnight cultures of *E. coli* MG1655 transformed with pBAD33 (control) and pBAD33_CdiA–CT^EC869^ were serially diluted, and the dilutions were spotted on LB agar plates containing 50 μg/ml chloramphenicol and supplemented with 1% (w/v) arabinose (lower panel) or 1% (w/v) glucose (upper panel). (**B**) Growth curves of *E. coli* MG1655 transformed with pBAD33 and pBAD33_CdiA–CT^EC869^. Induction of CdiA–CT^EC869^ suppresses the growth of *E. coli* harboring pBAD33_CdiA–CT^EC869^ in LB containing 50 μg/ml chloramphenicol. When the OD_660_ reached ∼0.3, arabinose was added (final concentration 0.02%) to the medium, and the culture was continued at 37°C. The arrow in the graph indicates the point of CdiA–CT^EC869^ induction by arabinose addition. (**C**) Schematic diagram of quantification and comparison of relative amounts of each aa-tRNA by LC/MS. (**D**) LC/MS analysis of RNase I–digested fragments of Ac-aa-tRNAs (or formyl-methionyl-tRNA_f_^Met^, fMet-A76) prepared from aa-tRNAs from *E. coli* with (pBAD33_CdiA–CT) or without (pBAD33, control) induction of CdiA–CT^EC869^. The amount of each Ac-aa-A76 derived from aa-tRNA prepared from cells with or without CdiA–CT^EC869^ induction is expressed as the amount relative to Ac-Ala-A76 in cells with or without induction of CdiA–CT^EC869^, respectively. (**E**) Change of relative amounts of each aa-tRNA (or fMet-tRNA_f_^Met^) in *E. coli* after CdiA–CT^EC869^ induction. The relative amounts of each aa-tRNA in *E. coli* with CdiA–CT^EC869^ induction were normalized against the amount of the corresponding aa-tRNA in *E. coli* without induction of CdiA–CT^EC869^. Error bars represent SDs of more than three independent experiments, and the data are presented as mean values ± SD. (**F**) Cleavage of each tRNA in *E. coli* was also evaluated by northern blotting of tRNAs (0.125 μg) prepared from *E. coli* with (+) or without (-) induction of CdiA–CT^EC869^.

### Specific aa-tRNAs are reduced by CdiA–CT^EC869^ expression *in vivo*

Our *in vitro* analyses suggested that CdiA–CT targets several tRNAs, including tRNA^Gln^, tRNA^Asn^, tRNA^Trp^ and tRNA_f_^Met^ (Figure 2E). The cleavage of tRNAs results in a shortage of aa-tRNAs and ultimately inhibits protein synthesis in the cell.

We analyzed the change in aa-tRNA levels upon expression of CdiA–CT in *E. coli*. CdiA–CT was expressed using the pBAD arabinose-inducible protein expression system. CdiA–CT expression in *E. coli* was toxic and suppressed cell growth on agar plates and in liquid media (Figure 3A, B). After induction of CdiA–CT in *E. coli*, we prepared aa-tRNAs under acidic conditions to prevent their deacylation. Immediately afterward, the α-NH_2_ groups of the aminoacyl bonds of aa-tRNAs were chemically acetylated by acetic anhydride, converting aa-tRNAs to stable acetyl aa-tRNAs (Ac-aa-tRNAs) (37). Finally, Ac-aa-tRNAs were hydrolyzed with RNase I, and the amounts of Ac-aa-A_76_ were quantified by LC/MS (Figure 3C) (32,33,38). Thus, the cellular levels of each aa-tRNA could be inferred from the amount of the corresponding Ac-aa-A76 fragment.

Because tRNA^Ala^ is not cleaved by CdiA–CT *in vitro* (Figure 2E) or *in vivo*, as described below, the relative amounts of individual aa-tRNAs with or without induction of CdiA–CT expression were normalized against the relative amount of Ala-tRNA^Ala^ with or without induction of CdiA–CT expression, respectively (Supplementary Figure S5). LC/MS analyses of RNase I–digested RNAs prepared from *E. coli* in which CdiA–CT expression had been induced demonstrated that the amounts of Ac-Gln-A_76_ (*m/z* = 438.17), Ac-Asn-A_76_ (*m/z* = 424.16), and Ac-Trp-A_76_ (*m/z* = 496.19) were reduced to <1–5% of those detected when CdiA–CT was not induced (Figure 3D, E). Thus, the amounts of Gln-tRNA^Gln^, Asn-tRNA^Asn^, and Trp-tRNA^Trp^ in cells were decreased by the action of CdiA–CT. Although the amount of Ac-Met-A_76_ (*m/z* = 441.15) was not altered by induction of CdiA–CT, the amount of formyl-Met-A_76_ (fMet-A_76_, *m/z* = 427.14) was reduced to ∼40% of that in the absence of CdiA–CT induction (Figure 3D, E). Thus, the amount of the initiator *N*-formyl-Met-tRNA_f_^Met^ was decreased by the action of CdiA–CT. These results are consistent with the *in vitro* data showing that in addition to tRNA^Gln^ and tRNA^Asn^, tRNA^Trp^ and tRNA_f_^Met^ were cleaved (Figure 2E). Furthermore, we prepared tRNAs from *E. coli* in which CdiA–CT expression had been induced and performed northern blots to monitor tRNA cleavage. Consistent with the *in vitro* analyses (Figure 2E) and *in vivo* aa-tRNA level analyses (Figure 3E), tRNA^Gln^, tRNA^Asn^, tRNA^Trp^ and tRNA_f_^Met^ were cleaved by the action of CdiA–CT *in vivo* (Figure 3F).

The reduction of the amounts of fMet-tRNA_f_^Met^*in vivo* was moderate (∼40%) relative to the reductions in the levels of Gln-tRNA^Gln^, Asn-tRNA^Asn^ and Trp-tRNA^Trp^ (Figure 3E). Consistent with this result, cleavage of intracellular tRNA_f_^Met^ was not as efficient as that of tRNA^Gln^, tRNA^Asn^, and tRNA^Trp^ (Figures 2E, 3F, Supplementary Figure S4E), because tRNA_f_^Met^ carries a less favorable A residue at position 73 (Figure 2D). The unique C1–A72 mismatch at the top of the acceptor stem of tRNA_f_^Met^ would be the main determinant of the cleavage by CdiA–CT. Taken together, these observations indicate that tRNA^Gln^, tRNA^Asn^ and tRNA^Trp^ are the primary targets of CdiA–CT. When these specific tRNAs are cleaved, protein synthesis should be blocked.

### Both uncharged and aminoacylated tRNAs are cleaved by CdiA–CT^EC869^ in the presence of translation factors

In living cells, more than ∼80% of tRNAs are aminoacylated, and the small amount of uncharged tRNAs resides in the ribosome E-site (46). The affinity between aa-tRNA and Tu:GTP is stronger than that between uncharged tRNA and Tu:GTP (47). Hence, we investigated whether aa-tRNA is more efficiently cleaved than tRNA (i.e. uncharged tRNA) by CdiA–CT in the presence of Tu, Ts, and GTP *in vitro*.

**Figure 4.**
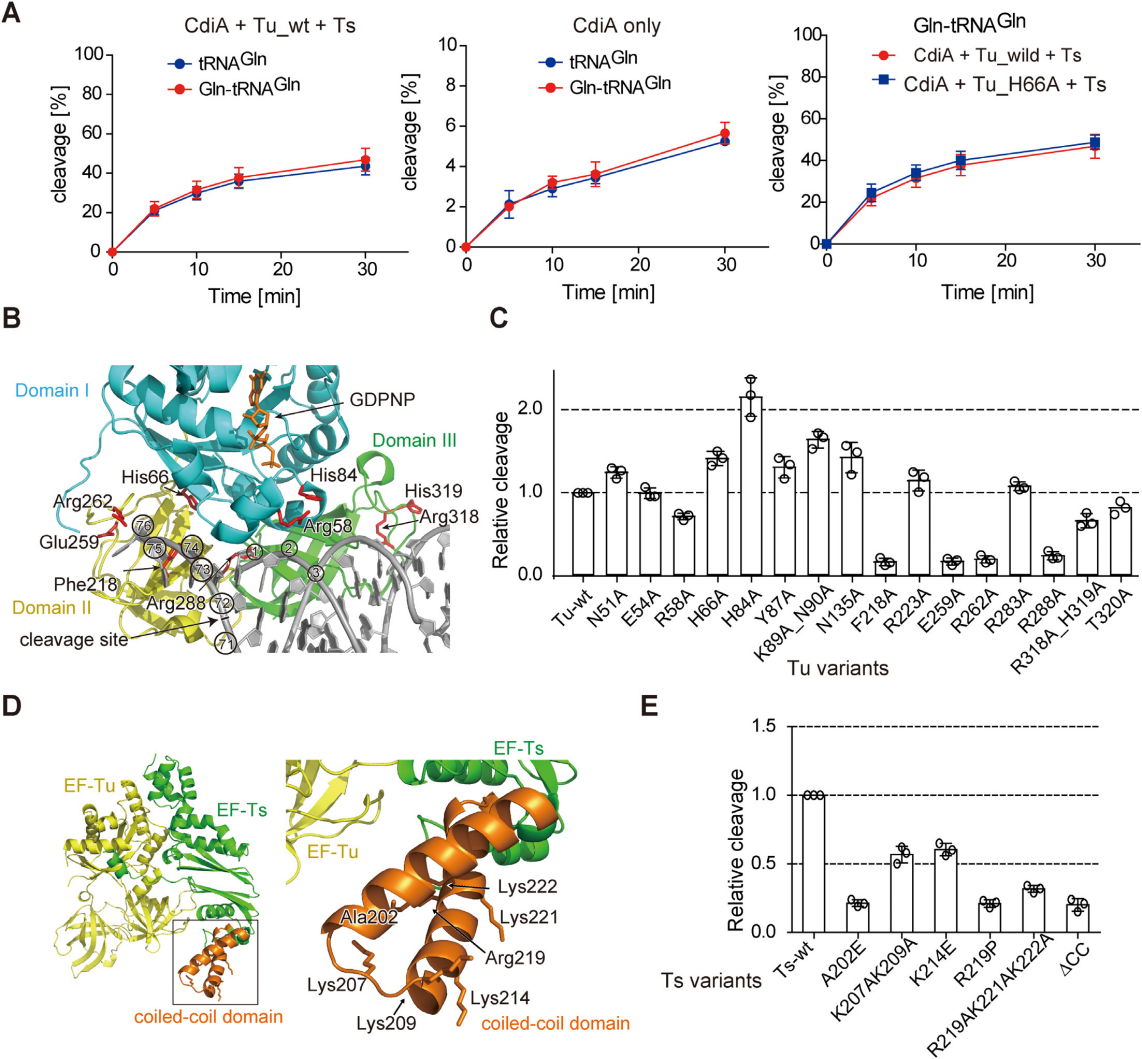
Involvement of RNA-binding residues in Tu or the coiled-coil domain of Ts. (**A**) Time courses of tRNA^Gln^ and Gln-tRNA^Gln^ cleavage by CdiA–CT^EC869^ in the presence (left) or absence (middle) of Tu, Ts, and GTP at 37°C. For cleavage of Gln-tRNA^Gln^, 1.0 μM of tRNA^Gln^ was aminoacylated by GlnRS (1.0 μM) for 60 min before cleavage by CdiA–CT^EC869^ (0.1 μM) in the absence or presence of translation factors (0.1 μM each) and GTP (1 mM) at pH 7.4. Under this condition, tRNA^Gln^ was aminoacylated to the level of 925 pmol/A_260_. Time courses of Gln-tRNA^Gln^ cleavage by CdiA–CT^EC869^ in the presence of wild-type or H66A mutant Tu (plus Ts, and GTP) (right). (**B**) Interaction of aa-tRNA with Tu (PDB ID: 1OB2). Domains I, II, and III of Tu are colored cyan, yellow, and green, respectively. Mutated residues are colored red. tRNA is shown as a gray stick model. The nucleotide numbers of tRNA are circled, and the site cleaved by CdiA–CT^EC869^ is depicted by an arrow. (**C**) Relative tRNA^Gln^ cleavage by CdiA–CT^EC869^ at 10 min in the presence of a Tu variant, Ts, and GTP. The tRNA^Gln^ transcript (1.0 μM) was incubated at 37°C for 10 min with 0.2 μM CdiA–CT^EC869^ in the presence of a Tu variant (0.2 μM), Ts (0.2 μM), and GTP (1 mM). (**D**) Crystal structure of the Tu:Ts complex (50). Tu and Ts are colored yellow and green, respectively. The coiled-coil domain in Ts is colored orange. (**E**) Relative tRNA^Gln^ cleavage by CdiA–CT^EC869^ at 10 min in the presence of Tu, a Ts variant, and GTP. The tRNA^Gln^ transcript (1.0 μM) was incubated at 37°C for 10 min with 0.2 μM CdiA–CT^EC869^ in the presence of Tu (0.2 μM), a Ts variant (0.2 μM), and GTP (1 mM). The tRNA^Gln^ cleavage level by CdiA–CT^EC869^ in the presence of Tu, Ts, and GTP was defined as 1.0 in (C) and (E). The bars in the graphs are SDs of more than three independent experiments, and the data are presented as mean values ± SD.

To this end, before CdiA–CT–mediated cleavage in the presence of Tu, Ts and GTP, tRNA^Gln^ was aminoacylated by glutamine-tRNA synthetase. Unexpectedly, prior aminoacylation of tRNAs did not significantly increase the tRNA cleavage level by CdiA–CT, irrespective of whether Tu, Ts, and GTP were present or absent (Figure 4A, left and middle graphs, Supplementary Figure S6). Thus, the aminoacyl moieties of substrate tRNAs are not required for the enhancement of tRNA cleavage by CdiA–CT in the presence of Tu, Ts, and GTP *in vitro*. To further verify this observation, instead of using wild-type Tu, a Tu variant with Ala substitution of His66 in domain I (H66A) was used in the assays. His66 interacts with the side chain of the aminoacyl moiety of aa-tRNAs in the Tu:GTP:aa-tRNA complex (48). The His66Ala mutation of Tu neither affected *in vitro* cleavage of Gln-tRNA^Gln^ (Figure 4A, right) nor that of deacylated tRNA^Gln^ as described below. These results indicate that both, aa-tRNAs and uncharged tRNAs, are targets of CdiA–CT *in vivo*. These results also imply that the enhancement of tRNA and aa-tRNA cleavage by CdiA–CT in the presence of translation factors does not proceed via Tu:GTP:aa-tRNA complex as described below.

### Involvement of Tu amino acid residues in tRNA cleavage

To evaluate whether the amino acid residues in Tu that are involved in the interactions with aa-tRNA in Tu:GTP:aa-tRNA complexes (Figure 4B) also participate in the promotion of tRNA cleavage by CdiA–CT, we prepared variant Tu proteins (Supplementary Figure S7A) and monitored the increase/decrease in tRNA cleavage by CdiA–CT in the presence of these variants (plus Ts and GTP). Ala substitutions of Phe218, Glu259 or Arg262 in domain II which interacts with the 3′-acceptor region of aa-tRNAs in Tu:GTP:aa-tRNA decreased the level of tRNA cleavage by CdiA–CT to that observed in the absence of Tu (Figure 4C). The Ala mutation of Arg288, which interacts with the 5′-phosphate of the tRNA in Tu:GTP:aa-tRNA, also decreased tRNA cleavage to the level observed in the absence of Tu (Figure 4C). However, Ala substitution of His66 in domain I, which interacts with the side chain of the aminoacyl moiety of an aa-tRNA in Tu:GTP:aa-tRNA, did not affect tRNA cleavage by CdiA–CT (Figure 4C). Mutations in Arg318 and His319 in domain III, which interact with the TΨC loop of tRNA, decreased the level of tRNA cleavage by CdiA–CT to ∼60% to that observed in the presence of wild-type Tu. Ala mutation of His84 in domain I, a catalytic residue of the GTPase center, increased the level of tRNA cleavage by CdiA–CT (Figure 4C). Tu with the H84A mutation might easily adopt the proper GTP-bound conformation required for enhancement of tRNA cleavage by CdiA–CT. Although the H84A mutation increased the tRNA cleavage level by CdiA–CT, GTP and Ts were still required for efficient cleavage of tRNA by CdiA–CT (Supplementary Figure S8). Ala mutation of Arg58 in domain I, which interacts with the phosphate of the nucleotide at position 2 of tRNA, decreased the RNA cleavage level by CdiA–CT to ∼60% of that in the presence of wild-type Tu (Figure 4C).

It should be noted that Tu mutations at residues (Phe218, Glu259, or Arg262) in domain II reduced the tRNA cleavage level by CdiA–CT (Figure 4C) in the presence of translation factors, while mutation of His66 in domain I did not. As described below, in the determined structure of CdiA–CT:Tu, these residues (Phe218, Glu259, or Arg262) in domain II of Tu are involved in the interaction between CdiA–CT and Tu, while His66 is not. Thus, the domain II mutations would decrease the tRNA cleavage level by CdiA–CT via reducing the interaction between Tu and CdiA–CT, rather than by decreasing the binding affinity of aa-tRNA (tRNA) to Tu. This may explain why tRNA cleavage by CdiA–CT in the presence of translation factors is independent of the aminoacyl status of the substrate tRNA (Figure 4A) and is not affected by the H66A mutation in domain I of Tu (Figure 4A, C).

### Involvement of the Ts coiled-coil domain in tRNA cleavage

Ts is a GEF for GDP-bound Tu that displaces the GDP and recycles Tu to the GTP form. Hence, we investigated whether the presence of excess Tu and GTP might promote tRNA cleavage by CdiA–CT, in which case Ts would no longer be required for promotion of cleavage. However, excess Tu (2.0 μM, 10-fold molar excess relative to 0.2 μM CdiA–CT) did not promote tRNA cleavage by CdiA–CT without Ts to the level observed in the presence of 0.2 μM Tu and 0.2 μM Ts (Supplementary Figure S9). Thus, Ts has a direct role in promoting tRNA cleavage by CdiA–CT together with Tu, rather than participating indirectly via its GEF function.

A recent study showed that the coiled-coil domain of Ts is required for the promotion of tRNA cleavage by CdiA–CT *in vitro* and that the Arg219Pro and Ala202Glu mutations in the coiled-coil domain inhibit the CdiA–CT^EC869^–mediated growth inhibition pathway *in vivo* (15). Notably, the coiled-coil domain contains several basic residues, Lys207, Lys209, Lys214, Arg219, Lys221 and Lys222 (Figure 4D, PDB ID: 4PC2). To evaluate the involvement of these basic residues in the promotion of tRNA cleavage by CdiA–CT, we prepared Ts variants (Supplementary Figure S7B). We examined the promotion of tRNA cleavage by CdiA–CT in the presence of the Ts variants (plus Tu and GTP). Triple Ala mutations at Arg219, Lys221 and Lys222 of Ts decreased the tRNA cleavage level by CdiA–CT to the same extent as Ala202Glu, Arg219Pro, or the coiled-coil deletion mutant (EF-Ts_ΔCC) (Figure 4E). The Lys207Ala–Lys209Ala and Lys214Glu Ts mutants also decreased tRNA cleavage level by CdiA–CT to ∼50% of the level in the presence of wild-type Ts. These results suggest that the basic residues in the coiled-coil domain of Ts are important for tRNA cleavage by CdiA–CT in the presence of translation factors and that the coiled-coil domain of Ts is involved in the interaction with tRNA during the reaction.

### Tethering of Ts to Tu promotes tRNA cleavage by CdiA–CT^EC869^

The results described above suggest that both Tu and Ts are likely to interact with tRNA during the tRNA cleavage by CdiA–CT, and we assumed that the Tu:GTP:Ts complex and CdiA–CT would interact with substrate tRNA during cleavage of substrate tRNAs.

**Figure 5.**
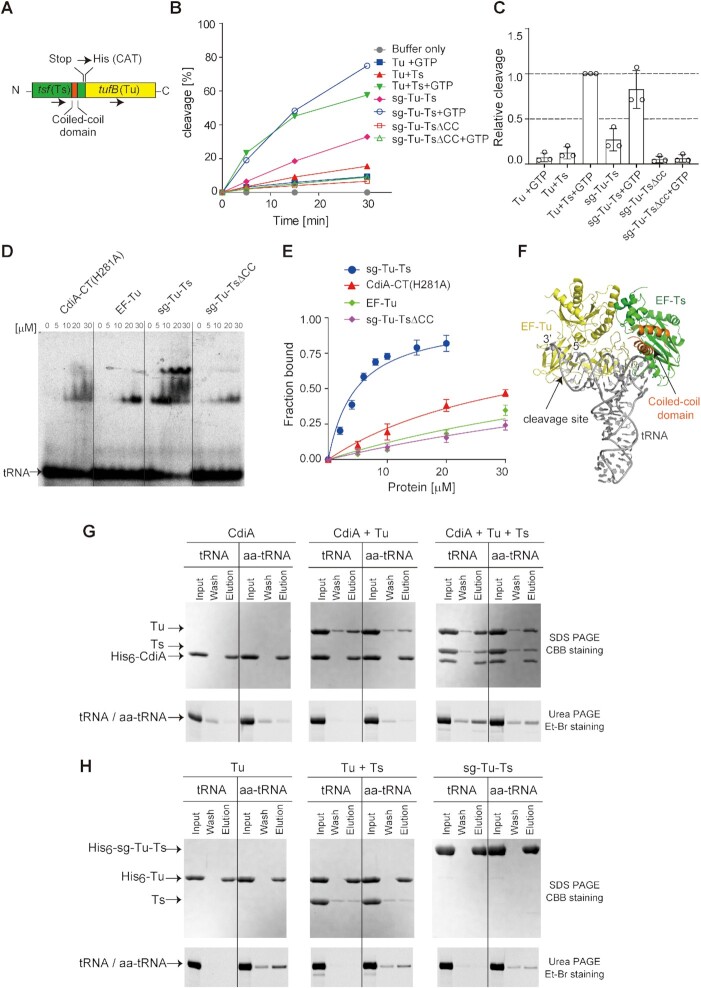
Formation of CdiA–CT:Tu:Ts:aa-tRNA (tRNA) in the presence of GTP. (**A**) Schematic diagram of single-chain Tu-Ts (sg–Tu–Ts). The stop codon of Ts gene (*tsf*) was changed to a histidine codon (CAT), and the Ts gene is followed by Tu (*tuf*B) gene in the same direction. (**B**) sg–Tu–Ts promotes tRNA^Gln^ cleavage by CdiA–CT. tRNA^Gln^ (1 μM) was incubated at 37°C with 0.1 μM CdiA–CT in the presence of Tu (0.1 μM), Ts (0.1 μM) or sg–Tu–Ts (0.1 μM) and GTP (1 mM). The fraction of cleaved tRNA was quantified at the indicated time points. (**C**) Relative tRNA^Gln^ cleavage by CdiA–CT at 5 min in the presence of Tu and Ts or sg–Tu–Ts (sg–Tu–Ts_ΔCC) and GTP at 37°C as in (B). The tRNA^Gln^ cleavage level by CdiA–CT^EC869^ in the presence of wild-type Tu, Ts and GTP was defined as 1.0. (**D**) Gel-shifts of tRNA^Gln^ by various amounts of CdiA–CT, Tu, sg–Tu–Ts, and sg–Tu–Ts_ΔCC (0–30 μM). The two complexes observed in the binding of sg–Tu–Ts to tRNA represent alternative binding modes. (**E**) tRNA fraction bound to each protein in (D). (**F**) A model of tRNA docking onto the Tu:GDPNP:Ts complex (50). Tu and Ts are colored yellow and green, respectively. The coiled-coil domain of Ts is colored orange and the tRNA is colored gray. The Tu:GTP:aa-tRNA structure (PDB ID: 1OB2) was superimposed onto the structure of Tu:GDPNP:Ts (PDB ID: 4PC7). For clarity, Tu:GTP in Tu:GTP:aa-tRNA is omitted. (**G**) His-tagged CdiA–CT was mixed with Tu or Tu and Ts in the presence of tRNA or aa-tRNA and GTP, and the mixture was loaded onto Ni-NTA column. The column was washed, and finally, CdiA–CT was eluted from the column. The quaternary complex of CdiA–CT:Tu:Ts:tRNA(aa-tRNA) was formed. Proteins were visualized by SDS PAGE stained with CBB, and tRNA was visualized by PAGE stained with ethidium bromide. (**H**) His-tagged Tu was mixed with aa-tRNA or tRNA in the absence (left) or presence (middle) of Ts and GTP, and the mixture was loaded onto a Ni-NTA column. The column was washed, and finally, Tu was eluted from the column. His-tagged sg–Tu–Ts was also mixed with aa-tRNA or tRNA in the same manner, and sg–Tu–Ts was eluted from the column (right). Proteins and RNA were visualized as in (G). The error in the graphs in (C) and (E) represent SDs of at least three independent experiments, and data are presented as average values ± SD.

We examined whether artificial tethering of Ts to Tu promotes tRNA cleavage by CdiA–CT. To this end, we fused Ts with Tu and prepared single-chain Tu-Ts (sg–Tu–Ts) (Figure 5A). sg–Tu–Ts was previously demonstrated to be functional in the Qβ replicase system (49). We then tested the promotion of tRNA cleavage by CdiA–CT in the presence of sg–Tu–Ts and GTP. In the presence of GTP, the sg–Tu–Ts increased the tRNA cleavage level by CdiA–CT to a very similar extent as in the presence of separate Tu and Ts (Figure 5B, C). By contrast, mutant sg–Tu–Ts lacking the coiled-coiled domain of Ts (sg–Tu–Ts_ΔCC) did not promote tRNA cleavage by CdiA–CT (Figure 5B, C). To evaluate the tRNA-binding affinities of, CdiA–CT, Tu, and sg–Tu–Ts, we performed gel-shift assays at room temperature (∼25°C). We used catalytically inactive CdiA–CT with H281A mutation, CdiA–CT (H281A), instead of using wild-type CdiA–CT, because tRNA could be cleaved during the pre-incubation of tRNA with wild-type CdiA–CT. sg–Tu–Ts efficiently interacted with tRNA, whereas sg–Tu–Ts_ΔCC did not (Figure 5D, E). The estimated *K*_d_ value of tRNA for sg–Tu–Ts was 4.7 μM, and the *K*_d_ value of tRNA for sg–Tu–Ts_ΔCC was >>30 μM (Figure 5E). The *K*_d_ value of tRNA for Tu was >>30 μM under the same condition. The *K*_d_ value of tRNA for mutant CdiA–CT (H281A) was >30 μM. sg–Tu–Ts is functionally equivalent to the Tu:Ts complex in terms of enhancement of tRNA cleavage by CdiA–CT *in vitro* (Figure 5B, C). These results suggest that the Tu:Ts complex promotes the tRNA cleavage activity of CdiA–CT in the presence of GTP, and that Ts interacting with Tu has a direct role in enhancement of tRNA cleavage by CdiA–CT.

In the Tu:GTP:Ts structure (50) (PDB ID: 4PC7), the tRNA-binding regions in Tu domains II and III are available for interactions with the acceptor stem of the tRNA, and the Tu binding sites for Ts and tRNA are not mutually exclusive. Previously, the structure of the Tu:GTP:aa-tRNA ternary complex (PDB ID: 1OB2) was superposed onto that of the Tu:GTP:Ts ternary complex, and the Tu:GTP:Ts:aa-tRNA complex was modeled (Figure 5F) (50). In the model, the coiled-coil domain of Ts is proximal to the elbow region of tRNA, explaining how Ts in Tu:GTP:Ts contributes to the interaction with aa-tRNA (tRNA).

### Formation of the CdiA–CT:Tu:Ts:aa-tRNA(tRNA) complex

To obtain further mechanistic insight into the requirement of both Tu and Ts in efficient cleavage of tRNAs by CdiA–CT, we performed pull-down assays to investigate whether Tu, Ts, and CdiA–CT simultaneously interact with tRNA^Gln^ (or Gln-tRNA^Gln^) (Figure 5G, Supplementary Figure S10).

Under the condition where aa-tRNA, but not uncharged tRNA, was pulled down with Tu in the presence of GTP (Figure 5H, left), CdiA–CT alone did not significantly pull-down tRNA or aa-tRNA (Figure 5G, left). Even in the presence of Tu and GTP, CdiA–CT did not pull-down tRNA or aa-tRNA, but did pull-down Tu (Figure 5G, middle), indicating that CdiA–CT:Tu:GTP:aa-tRNA (tRNA) is not formed. While only aa-tRNA, but not tRNA, was pulled down with Tu and Ts (or sg–Tu–Ts) (Figure 5H, middle and right), both tRNA and aa-tRNA were pulled down with CdiA–CT in the presence of Tu, Ts, and GTP with almost the same efficiency, together with Tu and Ts (Figure 5G, right). The interaction with tRNA was enhanced in the presence of GTP (Supplementary Figure S10B) and was dependent on the coiled-coil domain of Ts (Supplementary Figure S10C). Similar results were also obtained when sg–Tu–Ts and sg–Tu–Ts_ΔCC were used for pull-down assays. tRNA was pulled down with CdiA–CT and sg–Tu–Ts, but not with CdiA–CT and sg–Tu–Ts_ΔCC (Supplementary Figure S10D). Further, in the absence of tRNA, CdiA–CT pulled down Tu, but not Ts (15) (Supplementary Figure S10A), suggesting that CdiA–CT interacts with Tu even in the absence of tRNA.

Altogether, these observations suggest that binding of CdiA–CT to Tu:GTP:Ts cooperatively increases the aa-tRNA (tRNA) binding affinity and the quaternary complex of CdiA–CT:Tu:Ts:aa-tRNA(tRNA) can be efficiently formed in the presence of GTP. Notably, formation of the quaternary complex, CdiA–CT:Tu:Ts:aa-tRNA(tRNA), is independent of the aminoacyl status of tRNAs.

### Structure of CdiA–CT:CdiI^EC869^ in complex with Tu

To determine the molecular basis of the interaction between CdiA–CT and Tu, we crystallized CdiA–CT:CdiI^EC869^ in complex with Tu and determined the structure (Table 1, Supplementary Figure S11), as the CdiA–CT:CdiI^EC869^ complex interacts more tightly with Tu than CdiA–CT *in vitro* (Supplementary Figure S10A).

**Figure 6.**
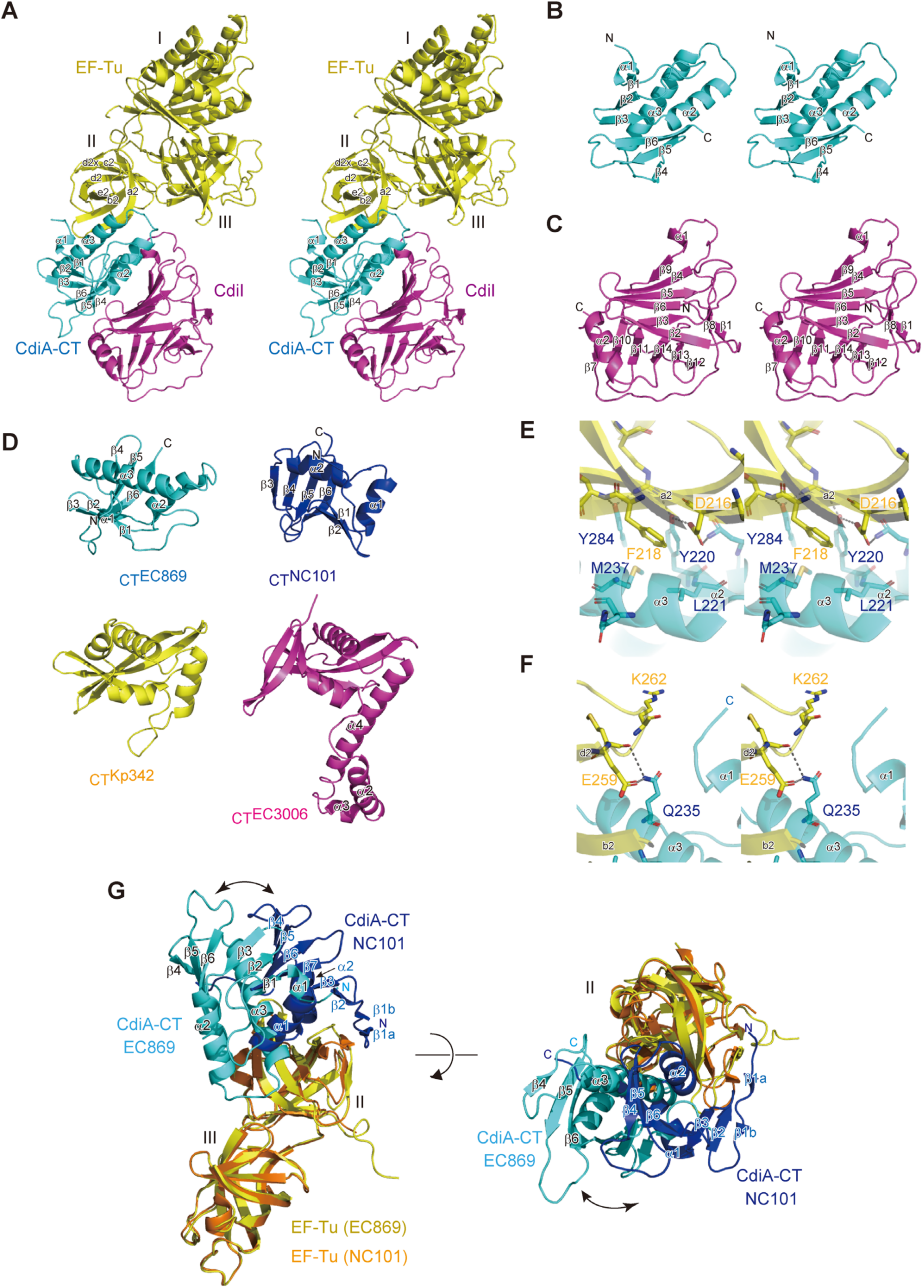
Structure of CdiA–CT:CdiI^EC869^ complexed with Tu. (**A**) Stereoview of the structure of the CdiA–CT:CdiI:Tu complex. CdiA–CT^EC869^, CdiI^EC869^ and Tu are colored cyan, magenta, and yellow, respectively. The modeled CdiA–CT^EC869^ contains residues 175–285 (numbered from Val1 of the VENN peptide motif, Supplementary Figure S13A), and the modeled CdiI^EC869^ contains residues 3–179. The modeled Tu contains residues 10–393. (**B**) Stereoview of CdiA–CT^EC869^. (**C**) Stereoview of CdiI^EC869^. (**D**) CdiA–CT^EC869^ adopts the BECR fold as observed in other CdiA–CTs targeting the 3′-acceptor region of tRNAs. CdiA–CT^NC101^ (blue) from *E. coli* NC101 (14), CdiA–CT^Kp342^ (yellow) from *Klebsiella pneumoniae*, and CdiA–CT^EC3006^ (magenta) from *E. coli* 3006 (16). (**E), (F**) Interaction between CdiA–CT (cyan) and domain II of Tu (yellow). (**G**) Superimposition of the structure of CdiA–CT^EC869^:Tu onto that of CdiA–CT^NC101^:Tu (14). For clarity, the structures of domain I of Tu in both complex structures are omitted. CdiA–CT^EC869^ and domains II/III of Tu in the structure of CdiA–CT^EC869^:Tu are colored cyan and yellow, respectively. CdiA–CT^NC101^ and domains II/III of Tu in the structure of CdiA–CT^NC101^:Tu are colored blue and orange, respectively.

In the structure of CdiA–CT:CdiI^EC869^ in complex with Tu, the CdiA–CT toxin interacts with domain II of Tu on one side and with CdiI on the other side (Figure 6A). The CdiA–CT toxin domain consists of an N-terminal three-stranded anti-parallel β-sheet (β1- β3) and a C-terminal three-stranded anti-parallel β-sheet (β4 - β6). These two β-sheets are flanked by two α-helices (α2 and α3). The N-terminal sheet interacts with the C-terminal sheet in a parallel orientation via β3 and β6, and the N-terminal and C-terminal sheets together wrap around α3 (Figure 6B). The CdiI^EC869^ protein consists of an N-terminal six-stranded mixed β-sheet (β2–β3−β6−β5−β4−β9; β4−β9 is parallel) and a C-terminal six-stranded mixed β-sheet (β7–β10–β11–β14–β13–β12; β7–β10 is parallel). These two are facing each other, thus adopting a β-sandwich structure (Figure 6C). The β-sandwich structure is supported by the surrounding anti-parallel β-sheet (β1–β8) and α-helix (α2).

The overall structure of the CdiA–CT toxin domain is topologically homologous to those of other CdiA toxin domains from *E. coli* 3006 (CdiA–CT^EC3006^), *E. coli* NC101 (CdiA–CT^NC101^) (14) and *Klebsiella pneumoniae* 342 (CdiA–CT^Kp342^) and adopts the Barnase/EndoU/Colicin/RelE (BECR) fold (16) (Figure 6D). All of these CdiA–CTs cleave the acceptor region of tRNAs (17). Although the structure of CdiA–CT^EC869^ is homologous to those of other CdiA–CTs with the BECR fold, the structures of the corresponding CdiIs are not homologous (Supplementary Figure S12A) (16). The interactions between CdiA–CT^EC869^ and CdiI^EC869^ are mainly mediated by hydrogen bonds (Supplementary Figure S12B, C). As described below, mutations His216 and His281 in CdiA–CT reduced the toxic activity of CdiA–CT *in vivo*, indicating that they are residues involved in catalysis and suggesting that CdiI inhibits the activity of CdiA–CT through masking the catalytic site of CdiA–CT, thereby preventing the 3′-portion of tRNA from binding to the catalytic pocket of CdiA–CT.

In this structure, Tu adopts the GDP-bound open form structure, although no GDP was bound to Tu. α3 of CdiA–CT is sandwiched between the β-barrel of the domain II of Tu and the β-sheet of CdiA–CT (Figure 6A). CdiA–CT extensively interacts with the domain II of Tu (Supplementary Figure S12D, E). In particular, Phe218 and Glu259 interact with CdiA–CT. Phe218 in Tu stacks with the side chain of Met237 and also interacts with Tyr220, Leu221 and Tyr284 in CdiA–CT through hydrophobic interactions, thereby stabilizing the CdiA–CT:Tu interaction (Figure 6E). Glu259 in Tu forms a hydrogen bond with the Nϵ atom of the side chain of Gln235 (Figure 6F). Arg262 in Tu might also interact with the N-terminal region of CdiA–CT, although the N-terminal region of CdiA–CT is unclear in the present structure. Reduced cleavage of tRNA by CdiA–CT in the presence of Tu mutants, such as Phe218Ala and Glu259Ala (Figure 4C), would be due to the reduced interaction between Tu and CdiA–CT, rather than reduced binding of aa-tRNA (tRNA) to Tu.

The structure of CdiA–CT^EC869^:Tu was superposed onto that of CdiA–CT^NC101^:Tu (14) (Figure 6G). Although CdiA–CT^NC101^ and CdiA–CT^EC869^ exhibit low similarity at the primary amino acid sequence level, they adopt the BECR fold (Figure 6D), and both interact with domain II of Tu (16). The shorter α helix (α2) in CdiA–CT^NC101^, which corresponds to α3 of CdiA–CT^EC869^, is more snugly accommodated in the space between the β-barrel of Tu domain II and the β-sheet of CdiA–CT^NC101^. Further, the N-terminal β-strands (β1a) in CdiA–CT^NC101^ additionally constitute a β-sheet with the β-strands of domain II of Tu, but the corresponding interactions are not observed between CdiA–CT^EC869^ and domain II of Tu. Thus, the two structures differ in terms of interaction modes of CdiA–CTs and Tu domain II, resulting in dissimilar position and orientation of the six-stranded ß-sheet surfaces containing the catalytic site of CdiA–CT, relative to the surface of Tu domain II (Figure 6G). These differences may be the cause for the differential effects the translation factors exert on tRNA cleavage by CdiA–CT^EC869^ versus CdiA–CT^NC101^.

### tRNA docking onto the CdiA–CT:Tu:Ts complex

**Figure 7.**
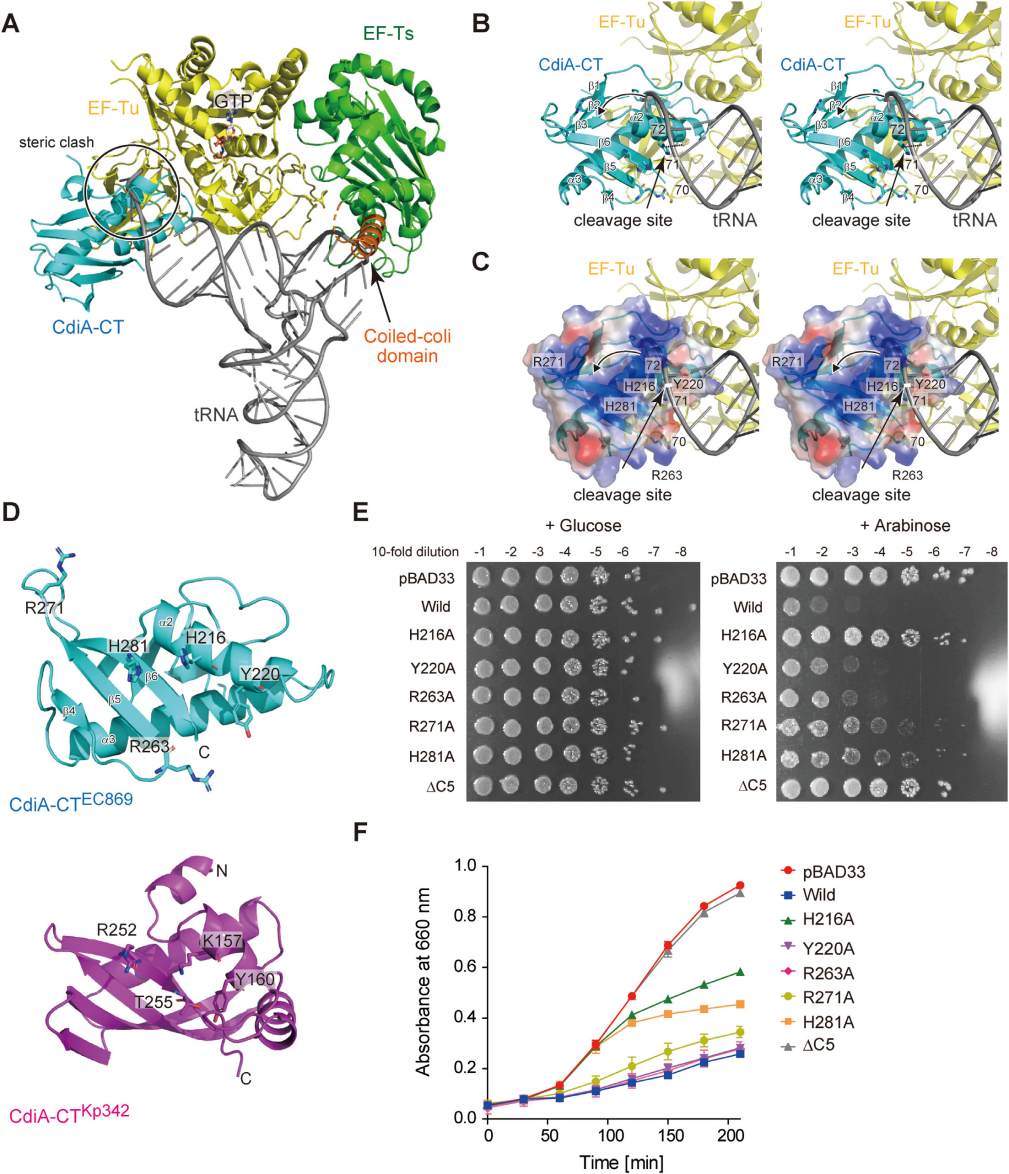
Recognition of the 3′-acceptor region of tRNA by CdiA–CT^EC869^. (**A**) A model of tRNA docking onto the CdiA–CT:Tu:GTP:Ts structure. CdiA–CT, Tu, and Ts are colored cyan, yellow, and green, respectively. The coiled-coil domain of Ts is colored orange. tRNA is shown in a stick model (gray). Domains II and III of Tu in the CdiA–CT:Tu complex structure were superimposed onto those in the Tu:GTP:Ts:aa-tRNA complex (Figure 5F), and the CdiA–CT:Tu:GTP:Ts:aa-tRNA model structure was constructed. (**B**) Close-up view of interactions of 3′-acceptor region of tRNA with CdiA–CT and Tu in (A). The 3′ portion of the tRNA sterically clashes with CdiA–CT. The arrowhead indicates the relocation of 3′-portion of tRNA into the catalytic site for tRNA cleavage. (**C**) The electrostatic surface potential of CdiA–CT. The positively charged area (blue) resides on the opposite side to the interface between Tu Domain II and CdiA–CT. (**D**) Comparison of structures of CdiA–CT^EC869^ and CdiA–CT^Kp342^ from *Klebsiella pneumoniae*. CdiA–CT^EC869^ and CdiA–CT^Kp342^ are colored cyan and magenta, respectively. The key amino acid residues required for toxicity and tRNase activity in CdiA–CT^Kp342^ (K157, Y160, R252 and T255) are shown as stick models (16). (**E**) Effects of mutations on toxicity of CdiA–CT^EC869^ expressed in *E. coli* MG1665, as in Figure 3A. LB agar plates containing 50 μg/ml chloramphenicol and supplemented with 0.5% (w/v) arabinose (right panel: +Arabinose) or with 1% (w/v) glucose (left panel: + Glucose). (**F**) Growth curves of *E. coli* MG1655 transformed with pBAD33, pBAD33_CdiA–CT^EC869^ and its variants in LB containing 50 μg/ml chloramphenicol and supplemented with 1% (w/v) arabinose.

We superimposed domains II and III of Tu in CdiA–CT:Tu complex structure onto those in the Tu:GTP:Ts:aa-tRNA complex and aa-tRNA was modeled onto the CdiA–CT:Tu:GTP:Ts structure (Figure 7A). In the superimposition, the 3′-acceptor part of the aa-tRNA sterically clashes with CdiA–CT. Thus, it is likely that the 3′-end of the aa-tRNA would adopt an alternative conformation to allow cleavage of the aa-tRNA between positions 71 and 72 by CdiA–CT (Figure 7B, C).

The electrostatic potential of the surface area of CdiA–CT in the CdiA–CT:Tu complex showed that the positively charged residues are clustered on the opposite side of the interface between CdiA–CT and Tu (Figure 7C). At the reaction stage of the CdiA–CT–mediated cleavage of tRNA between positions 71 and 72, the α2 helix in CdiA–CT may unwind the top of the acceptor stem of tRNA, and the loop between β1 and β2 would change its orientation, causing the 3′-end of tRNA substrate to relocate to the positively charged area of CdiA–CT (Figure 7C). The structure of CdiA–CT^EC869^ is homologous to that of CdiA–CT from *K. pneumoniae* 342 CdiA–CT^Kp342^ (Figure 6D), with an RMSD of 4.3 Å for 68 structurally equivalent residues (calculated by the Dali server (51); RMSD calculated with Cα atoms). In CdiA–CT^Kp342^, Lys157Ala, Tyr160Ala, Arg252Ala and Thr255Ala mutations decrease its toxicity and tRNase activity *in vivo* (16), and these residues are predicted to be involved in catalysis as well as substrate recognition. The residues His216 and Tyr220 in CdiA–CT^EC869^ would correspond to Lys157 and Tyr160 in CdiA–CT^Kp342^, respectively (Figure 7D). These residues would be proximal to the 3′-acceptor region of tRNA at the reaction stage (Figure 7B, C).

We examined whether the residues that contribute to the interaction with the 3′ region of the tRNA or catalysis are involved in the toxicity of CdiA–CT^EC869^ in *E. coli*. Mutation of His281 to alanine (H281A) or deletion of the C-terminal five amino acids (ΔC5, including H281; Supplementary Figure S13) decreased CdiA–CT^EC869^ toxicity when expressed in *E. coli* (Figure 7E, F). The substitution of His216 to Ala (H216A) or Arg271 to Ala (R271A) also decreased CdiA–CT^EC869^ toxicity. Arg263Ala and Tyr220Ala mutations did not decrease the toxicity significantly in liquid culture medium, but slightly attenuated the toxicity on agar plates (Figure 7E, F). These results are in line with the model that the 3′-acceptor region of tRNA interacts with the positively charged surface of CdiA–CT in cleavage-competent CdiA–CT:Tu:GTP:Ts:tRNA complexes. Although the catalytic residues involved in cleavage of tRNA between positions 71 and 72 have not been precisely identified, His216 and His281 are located proximal to the cleavage site in tRNA (Figure 7B, C) and might be involved in the catalysis.

## DISCUSSION

CdiA–CT^EC869^ has been reported to specifically cleave tRNA^Gln^ and tRNA^Asn^ in a reaction promoted by Tu, Ts, and GTP (Figure 1A) (15). In a recently proposed model of the requirement of translation factors for tRNA cleavage by CdiA–CT^EC869^ (14,15), CdiA–CT recognizes specific tRNAs in the context of the Tu:GTP:aa-tRNA complex, and Ts stimulates an increase in the steady-state level of Tu:GTP:aa-tRNA via formation of Tu:GTP:Ts:aa-tRNA complex, thereby facilitating recognition of Tu:GTP:aa-tRNA by CdiA–CT^EC869^ (14,52,53).

In this study, we showed that CdiA–CT^EC869^ itself has an intrinsic ability to select substrate tRNAs and cleave tRNA^Trp^ and tRNA_f_^Met^, in addition to the previously identified substrates tRNA^Gln^ and tRNA^Asn^, *in vitro* and *in vivo* (Figures 2E, 3E, F, Supplementary Figure S5). At neutral pH, both Tu and Ts are required for efficient tRNA cleavage by CdiA–CT^EC869^ (Figure 1). We also showed that the enhancement of tRNA cleavage by CdiA–CT^EC869^ in the presence of translation factors is independent of the aminoacyl status of tRNA, and that CdiA–CT^EC869^ cleaves uncharged tRNA and aa-tRNA to the same extent *in vitro* (Figure 4A, Supplementary Figure S6). In living cells, more than ∼80% of tRNAs are aminoacylated, and a small amount of uncharged tRNAs resides in the ribosome E-site (46). It is likely that primarily cleavage of aa-tRNAs, and to a smaller extent also cleavage of uncharged tRNAs, contribute to the CDI by CdiA–CT^EC869^*in vivo*.

We also showed sg–Tu–Ts, which tethers Ts to Tu and mimics the Tu:Ts complex, promotes tRNA cleavage by CdiA–CT^EC869^ to the same extent as in the presence of separate Tu and Ts proteins (Figure 5B). sg–Tu–Ts interacts with tRNA with a higher affinity than Tu alone, and the coiled-coil domain of Ts contributes to the higher affinity for tRNA (Figure 5E). Furthermore, CdiA–CT^EC869^ interacts with the domain II of Tu (Figure 6A) where the 3′-portion of aa-tRNA binds in Tu:GTP:aa-tRNA ternary complexes. CdiA–CT^EC869^, Tu, and Ts form a complex with tRNA and aa-tRNA in the presence of GTP *in vitro* (Figure 5G), and CdiA–CT:Tu:GTP:Ts:aa-tRNA (tRNA) complex formation is dependent on the coiled-coil domain of Ts (Supplementary Figure S10C, D).

Considering that cleavage of uncharged tRNA and aa-tRNA by CdiA–CT^EC869^ is enhanced by translation factors to the same extent, but aa-tRNA interacts with Tu:GTP and Tu:GTP:Ts with a much higher affinity than uncharged tRNA (Figure 5H), it is unlikely that tRNA cleavage by CdiA–CT in the presence of translation factors proceeds via formation of the Tu:GTP:aa-tRNA(tRNA) or Tu:GTP:Ts:aa-tRNA(tRNA) complex. Indeed, CdiA–CT^EC869^ interacts with the domain II of Tu where 3′-portion of aa-tRNA binds in Tu:GTP:aa-tRNA complex (Figure 6A).

**Figure 8.**
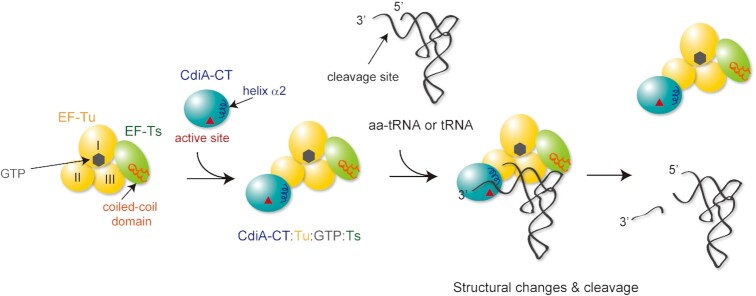
Model of tRNA cleavage by CdiA–CT^EC869^ in the presence of translation factors. The Tu:GTP:Ts complex acts as a scaffold for tRNA cleavage by CdiA–CT^EC869^. First, CdiA–CT^EC869^ delivered into the cell is recruited to the Tu:GTP:Ts complex to form the CdiA–CT:Tu:GTP:Ts complex. Substrate aa-tRNA (or tRNA) is recognized by CdiA–CT:Tu:GTP:Ts and forms CdiA–CT:Tu:GTP:Ts:aa-tRNA(tRNA). Ts in the CdiA–CT:Tu:GTP:Ts complex increases the affinity of tRNA for the complex and induces a structural change in tRNA and/or CdiA–CT to promote productive catalysis by CdiA–CT. CdiA–CT might interact with Tu:GTP and form CdiA–CT:Tu:GTP, and aa-tRNA (tRNA) could be recognized by the complex. However, aa-tRNA (tRNA) cannot be cleaved by CdiA–CT due to the low affinity of aa-tRNA (tRNA) to the CdiA–CT:Tu:GTP complex. Association of Ts to CdiA–CT:Tu:GTP or association of CdiA–CT to Tu:GTP:Ts is required for aa-tRNA (tRNA) binding and cleavage by CdiA–CT. It is not clear whether CdiA–CT first associates with Tu:GTP and then Ts is recruited to form CdiA–CT:Tu:GTP:Ts or CdiA–CT associates with Tu:GTP:Ts to form CdiA–CT:Tu:GTP:Ts.

Thus, tRNA cleavage proceeds via the CdiA–CT:Tu:GTP:Ts:tRNA(aa-tRNA) complex (Figure 8). CdiA–CT^EC869^ delivered into cells is recruited to domain II of Tu in the Tu:GTP:Ts complex and forms CdiA–CT:Tu:GTP:Ts. Then, tRNA and aa-tRNA could be recognized by the CdiA–CT:Tu:GTP:Ts complex. This model explains why the enhancement of tRNA cleavage by CdiA–CT is unaffected by mutation of His66, but is reduced by other Tu mutations in domain II (Phe218Ala, Glu259Ala, and Arg262Ala). Mutations of these Tu residues in domain II would reduce the interaction between Tu and CdiA–CT and thus reduce the enhancement of tRNA cleavage by CdiA–CT in the presence of translation factors. The interaction between CdiA–CT^EC869^ and the Tu:GTP:Ts complex would increase the affinity of the CdiA–CT:Tu:GTP:Ts complex for aa-tRNA (and tRNA). The coiled-coil domain of Ts in CdiA–CT:Tu:GTP:Ts enhances the affinity of aa-tRNA (tRNA) toward the complex and anchors the aa-tRNA (tRNA) substrate onto the complex. tRNA anchoring would prevent ejection of the tRNA from the complex, as the tail domains of CCA-adding enzymes do (54,55), during the structural shift of the 3′-region to the productive form that enables catalysis by CdiA–CT. According to the mechanistic model, α2 in CdiA–CT^EC869^ unwinds the top of the acceptor stem of tRNAs and, together with a conformational change of the loop between β1 and β2, induce the relocation of the 3′-part of tRNA into the active site of CdiA–CT^EC869^ for tRNA cleavage (Figure 7B, C).

The model proposed here also explains why tRNAs with a weak base pair or mismatch at the top of the acceptor stem are favorable substrates (Figure 2), whereas the mechanism underlying the requirement for G73 and pyrimidine and purine bases at positions 1 and 72 remains elusive. Under acidic conditions, CdiA–CT^EC869^ cleaves its specific substrate tRNAs without assistance of translation factors (Figure 1D, Supplementary Figure S2). Although the precise underlying mechanism remains unknown, under acidic conditions, the structure of the 3′-acceptor stem of tRNA or CdiA–CT might be altered, allowing CdiA–CT to cleave tRNAs more efficiently. The precise dynamical mechanisms underlying the promotion of tRNA cleavage by CdiA–CT in the presence of translation factors and the mechanisms of tRNA cleavage under acidic conditions by CdiA–CT alone await further structural determination of CdiA–CT in complex with translation factors and tRNAs.

CdiA–CT^EC869^, CdiA–CT^NC101^, CdiA–CT^EC3006^ and CdiA–CT^Kp342^ exhibit low similarity at the primary amino acid sequence level. However, the structures of CdiA–CT^NC101^, CdiA–CT^Kp342^, CdiA–CT^EC869^ and CdiA–CT^EC3006^ are homologous and adopt the BECR fold (Figure 6D), which includes the structure of the RNase domain of colicin D (16,56,57). CdiA–CT^EC869^, CdiA–CT^NC101^, CdiA–CT^EC3006^ and CdiA–CT^Kp342^ cleave specific tRNAs in their acceptor stems (14–17). CdiA–CT^NC101^ mainly cleaves tRNA^Asp^ and tRNA^Glu^ between nucleotide positions 72 and 73, CdiA–CT^Kp342^ cleaves tRNA^Ile^ between nucleotide positions 71 and 72, and CdiA–CT^EC3006^ cleaves tRNA^Ile^ between positions 70 and 71. For both CdiA–CT^NC101^ and CdiA–CT^Kp342^, as well as CdiA–CT^EC869^, specific tRNA cleavage is promoted in the presence of Tu, Ts, and GTP *in vitro* (14–16), and *tsf* mutants are resistant to growth inhibition by these CdiA–CTs. On the other hand, CdiA–CT^EC3006^ does not require Tu, Ts, or GTP for the cleavage of specific tRNAs, *in vitro* and possibly also *in vivo* (16). Notably, CdiA–CT^EC3006^ has an additional α-helical domain (α2, α3 and α 4) (Figure 6D). Perhaps the α-helical domain in CdiA–CT^EC3006^ interacts with the tRNA substrate together with the catalytic domain, allowing CdiA–CT^EC3006^ to efficiently cleave substrate tRNAs without the aid of translation factors (17).

As shown in this study, the cleavage of tRNAs by CdiA–CT^EC869^ is not affected by the aminoacyl status of substrate tRNA *in vitro* (Figure 4A); however, CdiA–CT^Kp342^ and CdiA–CT^EC3006^ cleave the 3′-acceptor region of uncharged tRNAs rather than aa-tRNAs *in vitro* (16). CdiA–CT^Kp342^ and CdiA–CT^EC3006^ target the small amount of uncharged tRNAs in the ribosome E-site (46) *in vivo*. The basic mechanism underlying tRNA cleavage by CdiA–CT^EC869^, CdiA–CT^NC101^ and CdiA–CT^Kp342^ in the presence of translation factors seems to be conserved. However, the specificity for certain tRNA substrates, the individual cleavage sites in the target tRNAs, and the recognition of the aminoacyl status of tRNA 3′-end are determined by the properties of the respective CdiA–CT variants. While the binding site for the 3′-moiety of tRNA on CdiA–CT^EC869^ is tolerant to the aminoacylation status, the corresponding binding sites for the tRNA 3′ -moiety on CdiA–CT^Kp342^ and CdiA–CT^EC3006^ are not. Thus, CdiA–CT^Kp342^ and CdiA–CT^EC3006^ cleave uncharged tRNAs, but not aa-tRNAs (16). The detailed molecular basis of substrate recognition by these CdiA–CTs awaits further structural determination of CdiA–CTs in complex with translation factors and tRNAs. Comparative analyses of CdiA–CTs that cleave 3′-acceptor regions of specific tRNAs will provide detailed views of the mechanism and evolution of these CdiA toxin families.

## DATA AVAILABILITY

Coordinates and structure factors for the crystal structure of CdiA–CT:CdiI^EC869^:Tu have been deposited to PDB under the accession number, 7VMC.

## Supplementary Material

gkac228_Supplemental_File
