# Supplementary material for: Mechanistic insights into tRNA cleavage by a contact-dependent growth inhibitor protein and translation factors
Source: Nucleic Acids Res. 2022 Apr 12;50(8):4713–31. doi: 10.1093/nar/gkac228 (PMC9071432; doi:10.1093/nar/gkac228)
Supplement: gkac228_Supplemental_File [file gkac228_supplemental_file.pdf]

Supplementary Information

Mechanistic insights into tRNA cleavage by a contact-dependent growth inhibitor protein and translation factors

Jing Wang, Yuka Yashiro, Yuriko Sakaguchi, Tsutomu Suzuki, Kozo Tomita

Figure S1

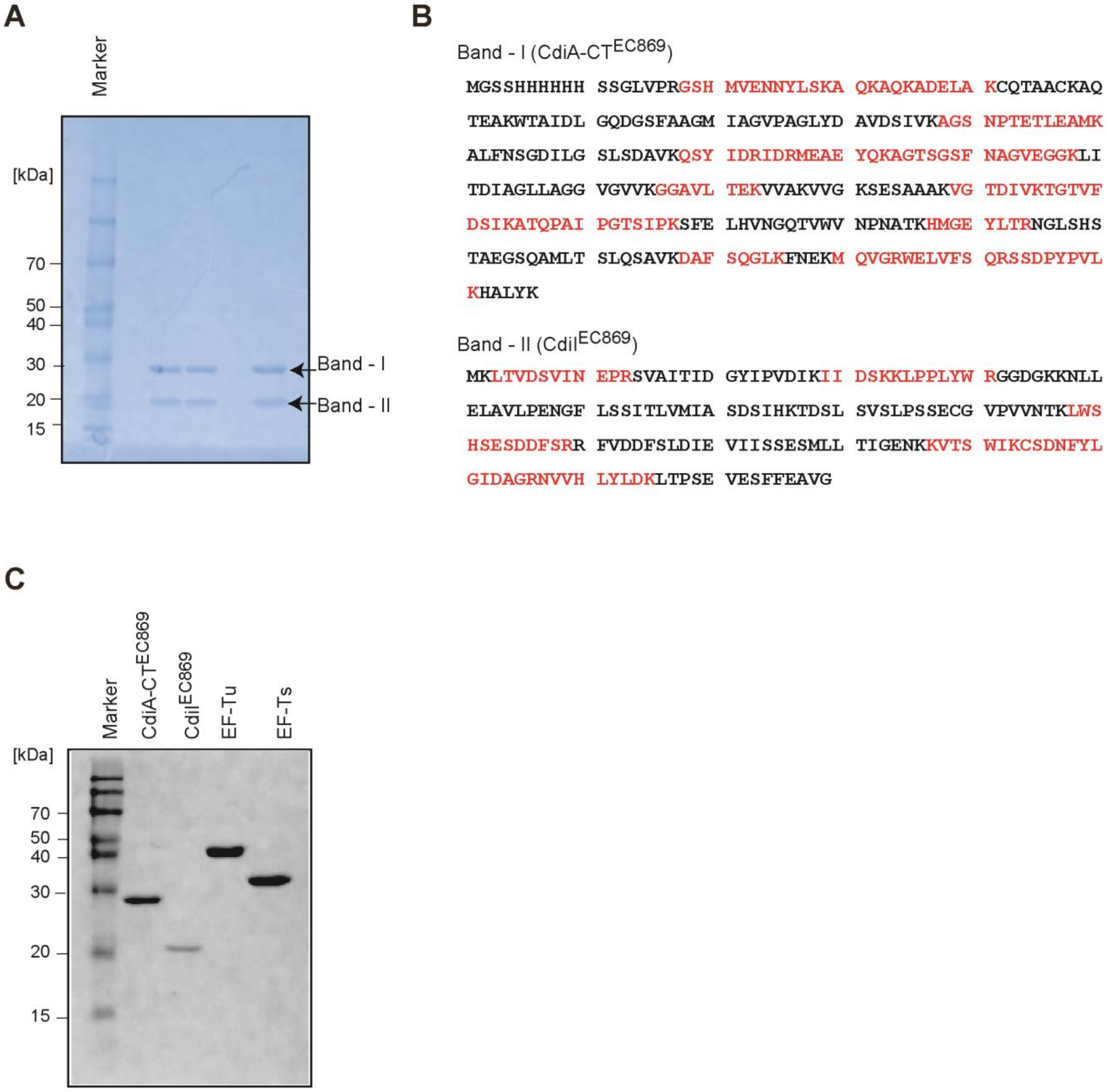

Supplementary Figure S1: Purification of recombinant proteins.

(**A**) Purification of the CdiA-CT^{EC869}:CdiI^{EC869} complex. After size-exclusion column chromatography, the fraction containing the CdiA-CT^{EC869}:CdiI^{EC869} complex was analyzed by electrophoresis on a 5–20% (w/v) SuperSep Ace gel (FujiFilm, Japan), which was stained with Quick-CBB Plus (Wako, Japan). Band-I and Band-II were digested with trypsin, and the peptides were analyzed by LC/MS, as described (1). (**B**) Peptides identified by LC/MS from the trypsin-digested Band-I and Band-II in (A) were mapped onto the amino acid sequences of recombinant CdiA-CT^{EC869} and CdiI^{EC869}, respectively. The amino acid sequences of recombinant CdiA-CT^{EC869} and CdiI^{EC869} are shown, and the identified peptide sequences are colored red. CdiI^{EC869} was co-purified with histidine-tagged CdiA-CT^{EC869}. (**C**) The purified Tu, Ts, CdiA-CT^{EC869}, and CdiI^{EC869} used for this study were separated by 15% (w/v) SDS PAGE, and the gel was stained with Coomassie Brilliant Blue.

Figure S2

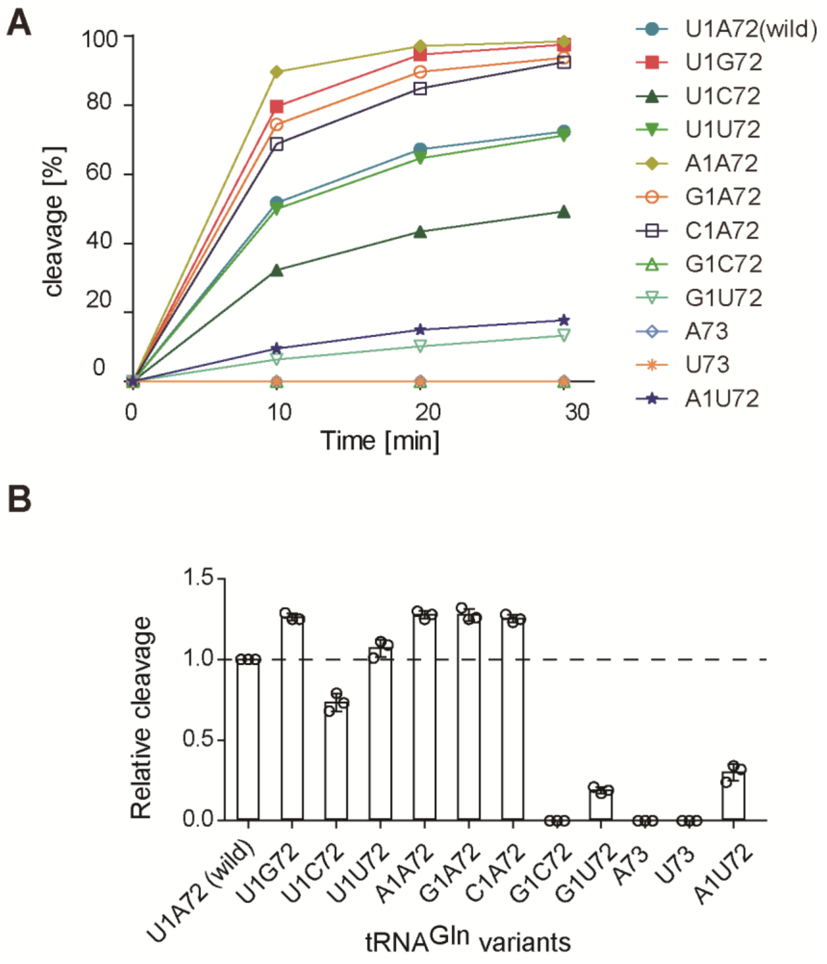

Supplementary Figure S2: Cleavage of tRNA^{Gln} variants by CdiA-CT^{EC869} under acidic conditions. (**A**) Time course of cleavage of tRNA^{Gln} variants by CdiA-CT^{EC869} under lower-pH conditions (pH 5.7) in the absence of Tu, Ts, and GTP. The tRNA^{Gln} variants (1.0 μM) were incubated with 0.1 μM of CdiA-CT^{EC869} at 37°C. The fraction of cleaved tRNA was quantified at the indicated time points. (**B**) The cleavage of tRNA^{Gln} variants by CdiA-CT^{EC869} in the absence of Tu, Ts, and GTP. The tRNA^{Gln} transcript variants (1.0 μM) were incubated with 0.1 μM of CdiA-CT^{EC869} at 37°C for 10 min. The cleavage level of wild-type tRNA^{Gln} (U1A72) was defined as 1.0. The bars in the graphs are SDs of more than three independent experiments, and the data are presented as mean values ± SD.

Figure S3

Amino Acid		Acc-stem		D-stem	D-loop	D-stem		Ac-stem	Ac-loop	Ac-stem	V-region	T-stem	T-loop	T-stem	Acc-stem		CCA
	-1	1	8	10	14	22	26	27	32	39	44	49	53	61	66	73	74
Asn_GTT	-	TCCTCTG	TA	GTTC	AGTC-GGT--A	GAAC	G	GCGGA	CTGTTAA	TCCGT	AT------------------GTC	ACTGG	TTCGAGT	CCAGT	CAGAGGA	G	CCA
Gln_CTG	-	TGGGGTA	TC	GCCA	AGC--GGT--A	AGGC	A	CCGGA	TTCTGAT	TCCGG	CA------------------TTC	CGAGG	TTCGAAT	CCTCG	TACCCCA	G	CCA
Gln_TTG	-	TGGGGTA	TC	GCCA	AGC--GGT--A	AGGC	A	CCGGT	TTTTGAT	ACCGG	CA------------------TTC	CCTGG	TTCGAAT	CCAGG	TACCCCA	G	CCA
Ini_CAT	-	CGCGGGG	TG	GAGC	AGCCTGGT--A	GCTC	G	TCGGG	CTCATAA	CCCGA	AG------------------ATC	GTCGG	TTCAAAT	CCGGC	CCCCGCA	A	CCA
Ini_CAT	-	CGCGGGG	TG	GAGC	AGCCTGGT--A	GCTC	G	TCGGG	CTCATAA	CCCGA	AG------------------GTC	GTCGG	TTCAAAT	CCGGC	CCCCGCA	A	CCA
Trp_CCA	-	AGGGGCG	TA	GTTC	AATT-GGT--A	GAGC	A	CCGGT	CTCCAAA	ACCGG	GT------------------GTT	GGGAG	TTCGAGT	CTCTC	CGCCCCT	G	CCA
Ala_GGC	-	GGGGCTA	TA	GCTC	AGCT-GGG--A	GAGC	G	CTTGC	ATGGCAT	GCAAG	AG------------------GTC	AGCGG	TTCGATC	CCGCT	TAGCTCC	A	CCA
Ala_TGC	-	GGGGCTA	TA	GCTC	AGCT-GGG--A	GAGC	G	CCTGC	TTTGCAC	GCAGG	AG------------------GTC	TGCGG	TTCGATC	CCGCA	TAGCTCC	A	CCA
Arg_ACG	-	GCATCCG	TA	GCTC	AGCT-GGAT-A	GAGT	A	CTCGG	CTACGAA	CCGAG	CG------------------GTC	GGAGG	TTCGAAT	CCTCC	CGGATGC	A	CCA
Arg_CCG	-	GCGCCCG	TA	GCTC	AGCT-GGAT-A	GAGC	G	CTGCC	CTCCGGA	GGCAG	AG------------------GTC	TCAGG	TTCGAAT	CCTGT	CGGGCGC	G	CCA
Arg_CCT	-	GTCCTCT	TA	GTTA	AAT--GGAT-A	TAAC	G	AGCCC	CTCCTAA	GGGCT	AA------------------T-T	GCAGG	TTCGATT	CCTGC	AGGGGAC	A	CCA
Arg_TCT	-	GCGCCCT	TA	GCTC	AGTT-GGAT-A	GAGC	A	ACGAC	CTTCTAA	GTCGT	GG------------------GCC	GCAGG	TTCGAAT	CCTGC	AGGGCGC	G	CCA
Asp_GTC	-	GGAGCGG	TA	GTTC	AGTC-GGTT-A	GAAT	A	CCTGC	CTGTCAC	GCAGG	GG------------------GTC	GCGGG	TTCGAGT	CCCGT	CCGTTCC	G	CCA
Cys_GCA	-	GGCGCGT	TA	ACAA	AGC--GGT--T	ATGT	A	GCGGA	TTGCAAA	TCCGT	CT------------------A-G	TCCGG	TTCGACT	CCGGA	ACGCGCC	T	CCA
Glu_TTC	-	GTCCCCT	TC	GTCT	AGA--GGCCCA	GGAC	A	CCGCC	CTTTCAC	GGCGG	TA------------------A-C	AGGGG	TTCGAAT	CCCCT	AGGGGAC	G	CCA
Gly_CCC	-	GCGGGCG	TA	GTTC	AAT--GGT--A	GAAC	G	AGAGC	TTCCCAA	GCTCT	AT------------------A-C	GAGGG	TTCGATT	CCCTT	CGCCCGC	T	CCA
Gly_GCC	-	GCGGGAA	TA	GCTC	AGTT-GGT--A	GAGC	A	CGACC	TTGCCAA	GGTCG	GG------------------GTC	GCGAG	TTCGAGT	CTCGT	TTCCCGC	T	CCA
Gly_TCC	-	GCGGGCA	TC	GTAT	AAT--GGCT-A	TTAC	C	TCAGC	CTTCCAA	GCTGA	TG------------------A-T	GCGGG	TTCGATT	CCCGC	TGCCCGC	T	CCA
His_GTG	G	GTGGCTA	TA	GCTC	AGTT-GGT--A	GAGC	C	CTGGA	TTGTGAT	TCCAG	TT------------------GTC	GTGGG	TTCGAAT	CCCAT	TAGCCAC	C	CCA
Ile_GAT	-	AGGCTTG	TA	GCTC	AGGT-GGTT-A	GAGC	G	CACCC	CTGATAA	GGGTG	AG------------------GTC	GGTGG	TTCAAGT	CCACT	CAGGCCT	A	CCA
Ile_CAT	-	GGCCCCT	TA	GCTC	AG-T-GGTt-A	GAGC	A	GGCGA	CTCATAA	TCGCT	TG------------------GTC	GCTGG	TTCAAGT	CCAGC	AGGGGCC	A	CCA
Ile_CAT	-	GGCCCTT	TA	GCTC	AG-T-GGTt-A	GAGC	A	GGCGA	CTCATAA	TCGCT	TG------------------GTC	GCTGG	TTCAAGT	CCAGC	AAGGGCC	A	CCA
Leu_CAA	-	GCCGAAG	TG	GCGA	AATC-GGTA-G	ACGC	A	GTTGA	TTCAAAA	TCAAC	CGTA-----GAAA-----TACGT	GCCGG	TTCGAGT	CCGGC	CTTCGGC	A	CCA
Leu_CAG	-	GCGAAGG	TG	GCGG	AATT-GGTA-G	ACGC	G	CTAGC	TTCAGGT	GTTAG	TGTTC----TTAC----GGACGT	GGGGG	TTCAAGT	CCCCC	CCCTCGC	A	CCA
Leu_CAG	-	GCGAAGG	TG	GCGG	AATT-GGTA-G	ACGC	G	CTAGC	TTCAGGT	GTTAG	TGTCC----TTAC----GGACGT	GGGGG	TTCAAGT	CCCCC	CCCTCGC	A	CCA
Leu_GAG	-	GCCGAGG	TG	GTGG	AATT-GGTA-G	ACAC	G	CTACC	TTGAGGT	GGTAG	TGCCC----AATA----GGGCTT	ACGGG	TTCAAGT	CCCGT	CCTCGGT	A	CCA
Leu_TAA	-	GCCCGGA	TG	GTGG	AATC-GGTA-G	ACAC	A	AGGGA	TTTAAAA	TCCCT	CGGCG----TTCG----CGCTGT	GCGGG	TTCAAGT	CCCGC	TCCGGGT	A	CCA
Leu_TAG	-	GCGGGAG	TG	GCGA	AATT-GGTA-G	ACGC	A	CCAGA	TTTAGGT	TCTGG	CGCC-----GCAA-----GGTGT	GCGAG	TTCAAGT	CTCGC	CTCCCGC	A	CCA
Lys_TTT	-	GGGTCGT	TA	GCTC	AGTT-GGT--A	GAGC	A	GTTGA	CTTTTAA	TCAAT	TG------------------GTC	GCAGG	TTCGAAT	CCTGC	ACGACCC	A	CCA
Met_CAT	-	GGCCCCT	TA	GCTC	AGT--GGTT-A	GAGC	A	GGCGA	CTCATAA	TCGCT	TG------------------GTC	GCTGG	TTCAAGT	CCAGC	AGGGGCC	A	CCA
Met_CAT	-	GGCCCTT	TA	GCTC	AGT--GGTT-A	GAGC	A	GGCGA	CTCATAA	TCGCT	TG------------------GTC	GCTGG	TTCAAGT	CCAGC	AAGGGCC	A	CCA
Met_CAT	-	GGCTACG	TA	GCTC	AGTT-GGTT-A	GAGC	A	CATCA	CTCATAA	TGATG	GG------------------GTC	ACAGG	TTCGAAT	CCCGT	CGTAGCC	A	CCA
Phe_GAA	-	GCCCGGA	TA	GCTC	AGTC-GGT--A	GAGC	A	GGGGA	TTGAAAA	TCCCC	GT------------------GTC	CTTGG	TTCGATT	CCGAG	TCCGGGC	A	CCA
Pro_CGG	-	CGGTGAT	TG	GCGC	AGCCTGGT--A	GCGC	A	CTTCG	TTCGGGA	CGAAG	GG------------------GTC	GGAGG	TTCGAAT	CCTCT	ATCACCG	A	CCA
Pro_GGG	-	CGGCACG	TA	GCGC	AGCCTGGT--A	GCGC	A	CCGTC	ATGGGGT	GTCGG	GG------------------GTC	GGAGG	TTCAAAT	CCTCT	CGTGCCG	A	CCA
Pro_TGG	-	CGGCGAG	TA	GCGC	AGCTTGGT--A	GCGC	A	ACTGG	TTTGGGA	CCAGT	GG------------------GTC	GGAGG	TTCGAAT	CCTCT	CTCGCCG	A	CCA
Ser_CGA	-	GGAGAGA	TG	CCGG	AGC--GGCTGA	ACGG	A	CCGGT	CTCGAAA	ACCGG	AGTAGGG--GCAA--CTCTAC-C	GGGGG	TTCAAAT	CCCCC	TCTCTCC	G	CCA
Ser_GCT	-	GGTGAGG	TG	GCCG	AGA--GGCTGA	AGGC	G	CTCCC	CTGCTAA	GGGAG	TATGCGGTCAAA-AGCTGCAT-C	CGGGG	TTCGAAT	CCCCG	CCTCACC	G	CCA
Ser_GGA	-	GGTGAGG	TG	TCCG	AGT--GGCTGA	AGGA	G	CACGC	CTGGAAA	GTGTG	TATACG---GCAA---CGTAT-C	GGGGG	TTCGAAT	CCCCC	CCTCACC	G	CCA
Ser_TGA	-	GGAAGTG	TG	GCCG	AGC--GGTTGA	AGGC	A	CCGGT	CTTGAAA	ACCGG	CGACCC---GAAA---GGGTT-C	CAGAG	TTCGAAT	CTCTG	CGCTTCC	G	CCA
Thr_CGT	-	GCTCAAG	TA	GTTA	AAAA-TGCA-T	TAAC	A	TCGCA	TTCGTAA	TGCGA	AG------------------GTC	GTAGG	TTCGACT	CCTAT	TATCGGC	A	CCA
Thr_CGT	-	GCCGATA	TA	GCTC	AGTT-GGT--A	GAGC	A	GCGCA	TTCGTAA	TGCGA	AG------------------GTC	GTAGG	TTCGACT	CCTAT	TATCGGC	A	CCA
Thr_GGT	-	GCTGATA	TG	GCTC	AGTT-GGT--A	GAGC	G	CACCC	TTGGTAA	GGGTG	AG------------------GTC	CCCAG	TTCGACT	CTGGG	TATCAGC	A	CCA
Thr_GGT	-	GCTGATA	TA	GCTC	AGTT-GGT--A	GAGC	G	CACCC	TTGGTAA	GGGTG	AG------------------GTC	GGCAG	TTCGAAT	CTGCC	TATCAGC	A	CCA
Thr_TGT	-	GCCGACT	TA	GCTC	AGTA-GGT--A	GAGC	A	ACTGA	CTTGTAA	TCAGT	AG------------------GTC	ACCAG	TTCGATT	CCGGT	AGTCGGC	A	CCA
Tyr_GTA	-	GGTGGGG	TT	CCCG	AGC--GGCCAA	AGGG	A	GCAGA	CTGTAAA	TCTGC	CGTC-----ATCG-----ACTTC	GAAGG	TTCGAAT	CCTTC	CCCCACC	A	CCA
Tyr_GTA	-	GGTGGGG	TT	CCCG	AGC--GGCCAA	AGGG	A	GCAGA	CTGTAAA	TCTGC	CGTC-----ACAG-----ACTTC	GAAGG	TTCGAAT	CCTTC	CCCCACC	A	CCA
Val_GAC	-	GCGTTCA	TA	GCTC	AGTT-GGTT-A	GAGC	A	CCACC	TTGACAT	GGTGG	GG------------------GTC	GTTGG	TTCGAGT	CCAAT	TGAACGC	A	CCA
Val_GAC	-	GCGTCCG	TA	GCTC	AGTT-GGTT-A	GAGC	A	CCACC	TTGACAT	GGTGG	GG------------------GTC	GGTGG	TTCGAGT	CCACT	CGGACGC	A	CCA
Val_TAC	-	GGGTGAT	TA	GCTC	AGCT-GGG--A	GAGC	A	CCTCC	CTTACAA	GGAGG	GG------------------GTC	GGCGG	TTCGATC	CCGTC	ATCACCC	A	CCA

Supplementary Figure S3: Sequence alignments of *E. coli* tRNA genes (2).

Figure S4

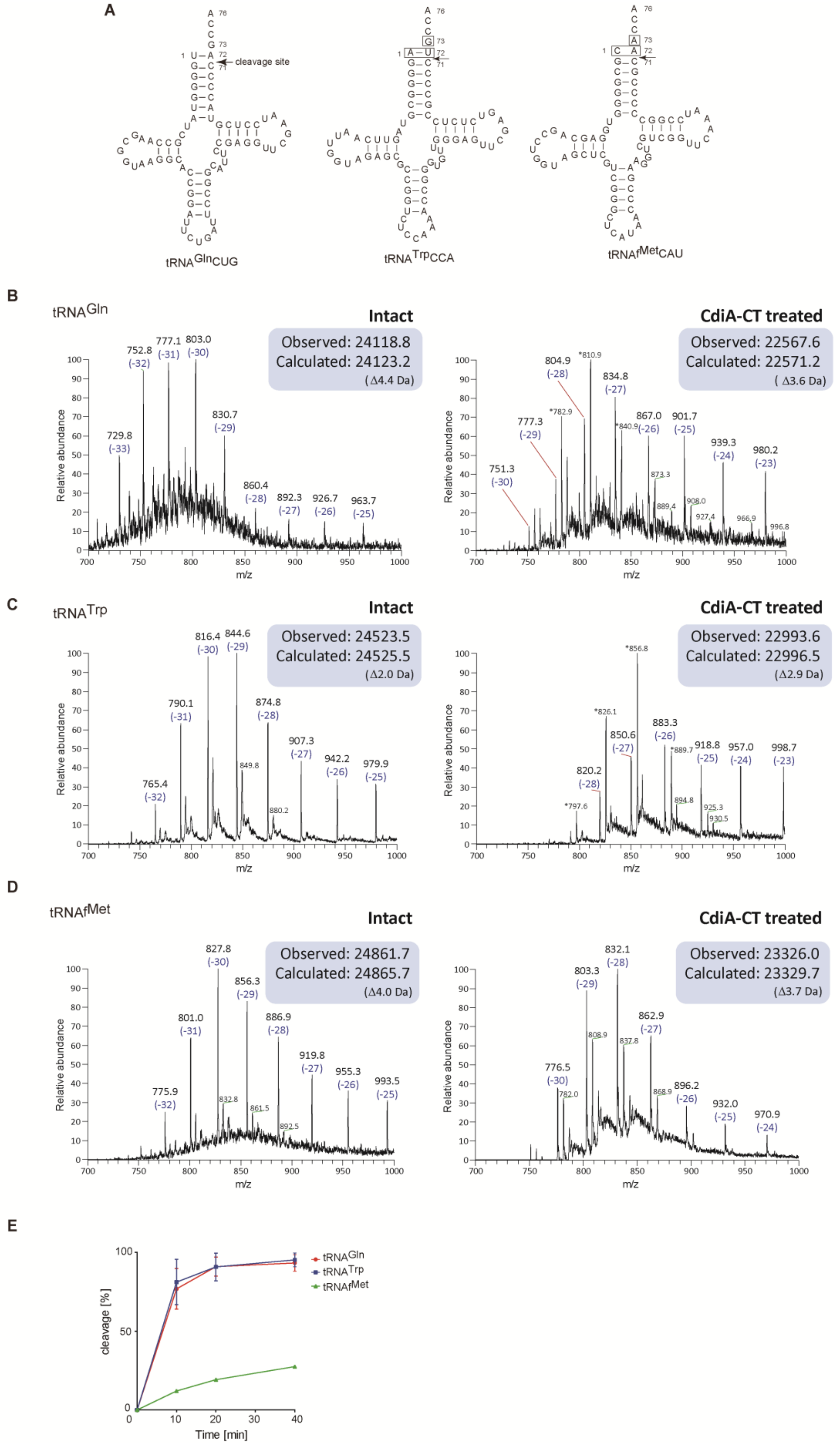
A
cleavage site
tRNAGlnCUG
tRNATrpCCA
tRNAfMetCAU
B
tRNAGln
Intact
Observed: 24118.8
Calculated: 24123.2
(Δ4.4 Da)
CdiA-CT treated
Observed: 22567.6
Calculated: 22571.2
(Δ3.6 Da)
C
tRNATrp
Intact
Observed: 24523.5
Calculated: 24525.5
(Δ2.0 Da)
CdiA-CT treated
Observed: 22993.6
Calculated: 22996.5
(Δ2.9 Da)
D
tRNAfMet
Intact
Observed: 24861.7
Calculated: 24865.7
(Δ4.0 Da)
CdiA-CT treated
Observed: 23326.0
Calculated: 23329.7
(Δ3.7 Da)
Relative abundance
m/z
E
cleavage [%]
Time [min]
tRNAGln
tRNATrp
tRNAfMet

Supplementary Figure S4: Cleavage sites in tRNA$_f^{Met}$ and tRNA^{Trp} by CdiA-CT^{EC869} *in vitro*.

(**A**) Clover-leaf structures of *E. coli* tRNA^{Gln}CUG, tRNA^{Trp}CCA, and tRNA$_f^{Met}$CAU. Cleavage sites in tRNAs are indicated by arrows. (**B**)-(**D**) Whole-mass analysis of CdiA-CT-treated tRNAs for Gln (B), Trp (C), and fMet (D). After tRNA cleavage by CdiA-CT in the presence of Tu, Ts and GTP, the cleaved products were separated by PAGE, purified, and analyzed. Left and right panels show ESI-MS of intact and CdiA-CT-treated tRNAs, respectively. A series of multiply charged negative ions (black) with charge values (blue) are shown in each mass spectrum. The deconvoluted molecular mass (Observed) and calculated value (Calculated) are shown in the inset. MS peaks of HFIP adducts are asterisked. The molecular mass reductions of tRNAs upon CdiA treatment indicated that these tRNAs are cleaved between positions 71 and 72, yielding terminal 2'-3' cyclic phosphate ends. (**E**) Time courses of the cleavage of tRNA^{Trp} and tRNA$_f^{Met}$ by CdiA-CT^{EC869} in the presence of Tu, Ts, and GTP at pH 7.4. The tRNAs (1.0 μM) were incubated with 0.6 μM of CdiA-CT^{EC869} at 37°C in the presence of 0.6 μM Tu, 0.6 μM Ts, and 1 mM GTP. The fraction of each cleaved tRNA was quantified at the indicated time points. The bars in the graphs are SDs of more than three independent experiments.

Figure S5

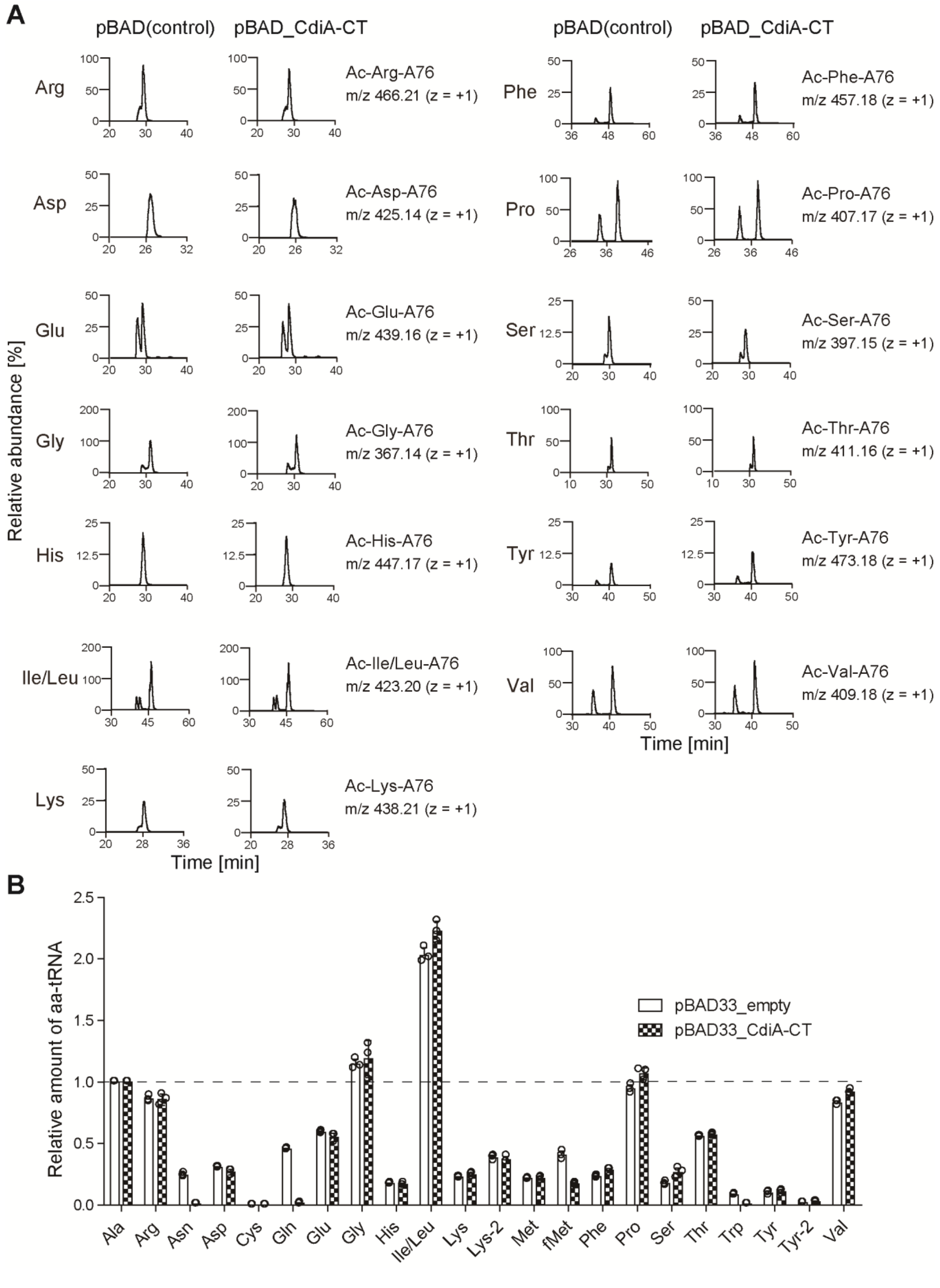
A
pBAD(control)
pBAD_CdiA-CT
Arg
Ac-Arg-A76
m/z 466.21 (z = +1)
Asp
Ac-Asp-A76
m/z 425.14 (z = +1)
Glu
Ac-Glu-A76
m/z 439.16 (z = +1)
Gly
Ac-Gly-A76
m/z 367.14 (z = +1)
His
Ac-His-A76
m/z 447.17 (z = +1)
Ile/Leu
Ac-Ile/Leu-A76
m/z 423.20 (z = +1)
Lys
Ac-Lys-A76
m/z 438.21 (z = +1)
Phe
Ac-Phe-A76
m/z 457.18 (z = +1)
Pro
Ac-Pro-A76
m/z 407.17 (z = +1)
Ser
Ac-Ser-A76
m/z 397.15 (z = +1)
Thr
Ac-Thr-A76
m/z 411.16 (z = +1)
Tyr
Ac-Tyr-A76
m/z 473.18 (z = +1)
Val
Ac-Val-A76
m/z 409.18 (z = +1)
Relative abundance [%]
Time [min]
B
Relative amount of aa-tRNA
pBAD33_empty
pBAD33_CdiA-CT
Ala Arg Asn Asp Cys Gln Glu Gly His Ile/Leu Lys Lys-2 Met fMet Phe Pro Ser Thr Trp Tyr Tyr-2 Val

Supplementary Figure S5: Changes of aa-tRNA levels after CdiA-CT^{EC869} expression.

(**A**) LC/MS analyses of RNase I–digested RNAs prepared from *E. coli* with or without CdiA-CT^{EC869} induction. The amount of each Ac-aa-A76 derived from the aminoacyl-tRNA prepared from cells, with or without induction of CdiA-CT^{EC869}, is expressed relative to the amount of Ac-Ala-A76 in each cell, with or without induction of CdiA-CT^{EC869}, respectively. (**B**) Quantification of relative amounts of each aa-tRNA (or formyl-Met-tRNA$_f^{Met}$: fMet) in *E. coli* with (pBAD33_CdiA-CT) or without (pBAD33_empty) induction of CdiA-CT^{EC869}. The amount of Ala-tRNA^{Ala} in each cell with or without CdiA-CT^{EC869} induction was set to 1.0. Lys-2 and Tyr-2 correspond to the products acetylated at the amino group and hydroxy group of the side chains, respectively, in addition to the α-amino groups. The bars in the graphs are SDs of more than three independent experiments.

Figure S6

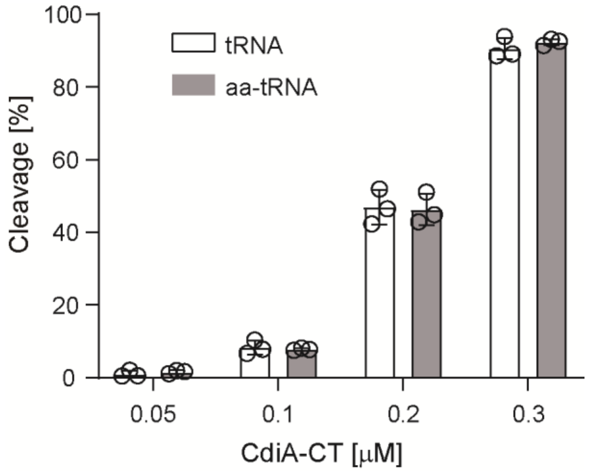

Supplementary Figure S6: Uncharged- and aminoacylated-tRNAs are cleaved by CdiA-CT^{EC869} in the presence of translation factors.

tRNA^{Gln} (tRNA, open box) and Gln-tRNA^{Gln} (aa-tRNA, closed box) cleavage by CdiA-CT^{EC869} in the presence of Tu, Ts, and GTP at 37°C. For cleavage of Gln-tRNA^{Gln}, 1.0 μM tRNA^{Gln} was first aminoacylated by GlnRS (1.0 μM) for 60 min in buffer containing 20 mM Tris-Cl, pH 7.4, 150 mM KCl, 5 mM $MgCl_2$, 10 mM β-mercaptoethanol, 4 mM ATP, and 200 μM glutamine, and was then cleaved by increasing amounts of CdiA-CT^{EC869} (0.05, 0.1, 0.2, and 0.3 μM), in the presence of translation factors (0.05, 0.1, 0.2, and 0.3 μM each) and GTP (1 mM) for 10 min. The tRNA cleavage levels were quantified. The bars in the graphs are SDs of more than three independent experiments.

Figure S7

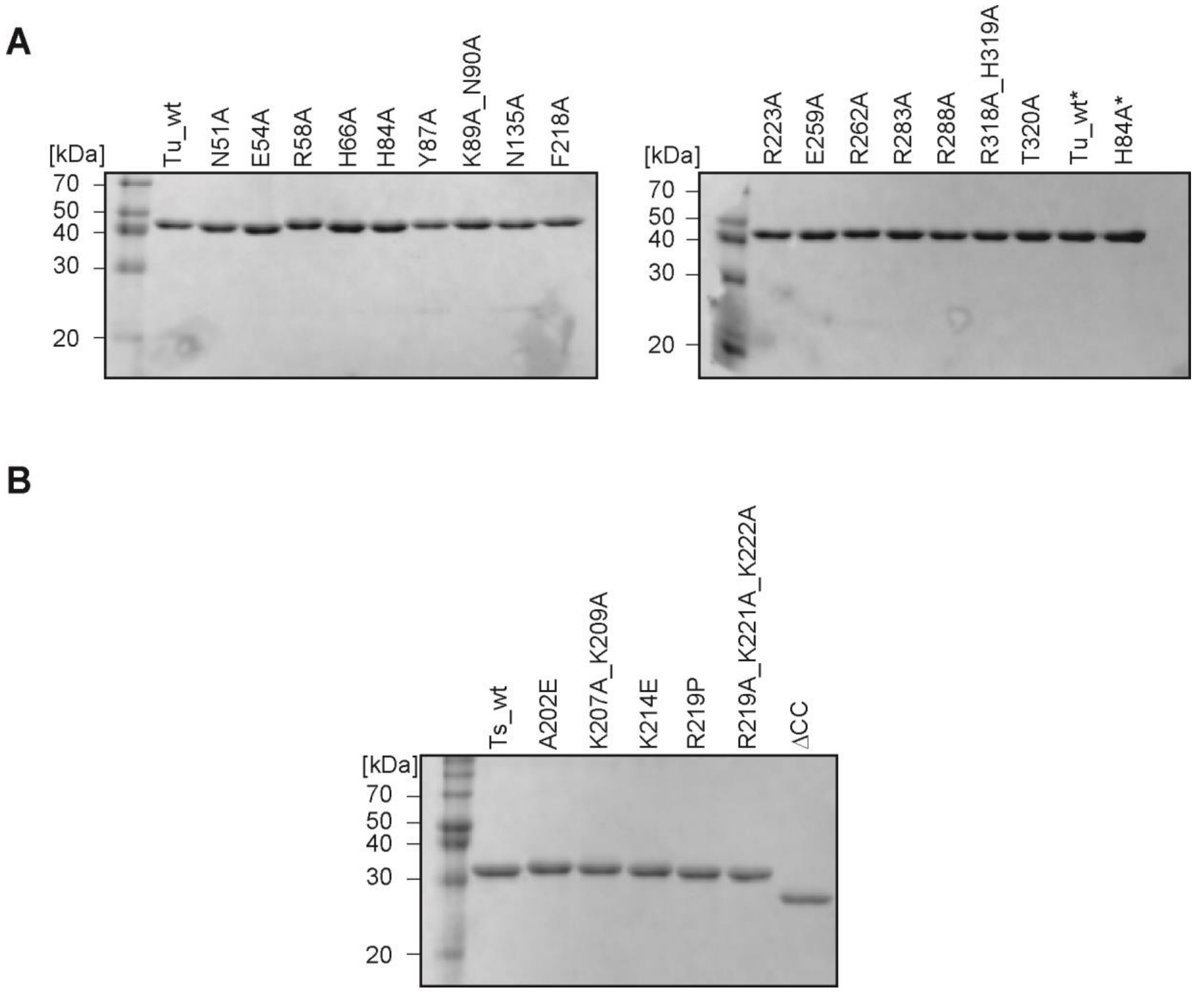

Supplementary Figure S7: Purification of Tu and Ts variants used in this study.
(**A**) Tu variants and (**B**) Ts variants were separated by 15% (w/v) SDS PAGE, and the gels were stained with Coomassie Brilliant Blue. Tu_wt* and H84A* in (A) were purified as previously described (3).

Figure S8

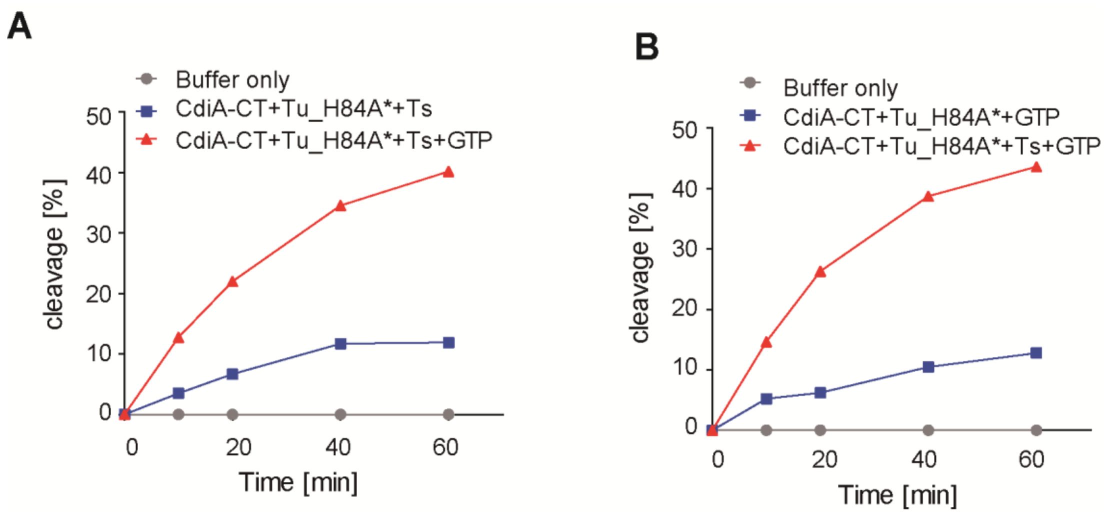

Supplementary Figure S8: The role of Tu_H84A in the enhancement of tRNA cleavage by CdiA-CT^{EC869}. (**A**) GTP requirement for the enhancement of tRNA cleavage by CdiA-CT^{EC869} in the presence of Tu_H84A and Ts. The tRNA^{Gln} transcript (1.0 μM) was incubated at 37°C with 0.1 μM of CdiA-CT^{EC869} in the presence of Tu_H84A (0.1 μM) and Ts (0.1 μM) with or without GTP (1 mM). (**B**) Ts requirement for the enhancement of tRNA cleavage by CdiA-CT^{EC869} in the presence of Tu_H84A and GTP. The tRNA^{Gln} transcript (1.0 μM) was incubated at 37°C with 0.1 μM of CdiA-CT^{EC869} in the presence of Tu_H84A (0.1 μM) and GTP (1 mM), with or without Ts (0.1 μM).

Figure S9

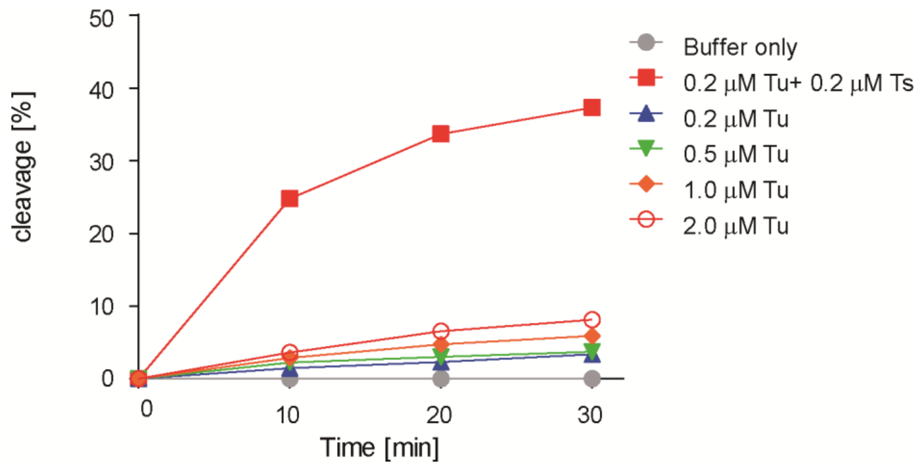

Supplementary Figure S9: Excess Tu does not compensate for the absence of Ts.
Cleavage of $tRNA^{Gln}$ by $CdiA\text{-}CT^{EC869}$ in the presence of increasing amounts of Tu and GTP, in the absence of Ts. The $tRNA^{Gln}$ transcript (1.0 μM) was incubated at 37°C with 0.2 μM of $CdiA\text{-}CT^{EC869}$ in the presence of Tu (0.2–2.0 μM) and GTP (1 mM). Addition of 0.2 μM Ts promoted cleavage of $tRNA^{Gln}$ at pH 7.4.

Figure S10

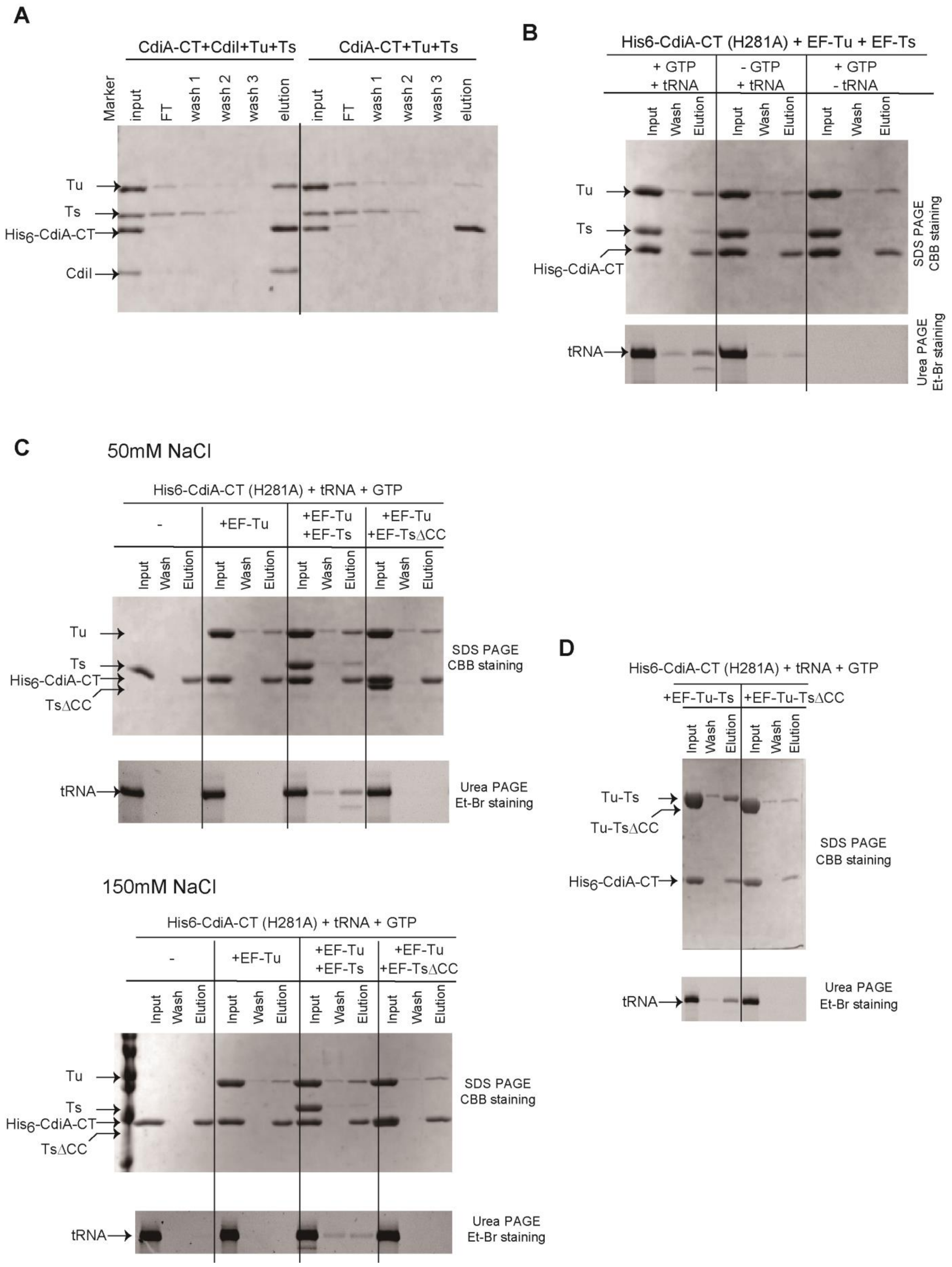
A
CdiA-CT+CdiI+Tu+Ts
CdiA-CT+Tu+Ts
Marker
input
FT
wash 1
wash 2
wash 3
elution
Tu
Ts
His6-CdiA-CT
CdiI
B
His6-CdiA-CT (H281A) + EF-Tu + EF-Ts
+ GTP + tRNA
- GTP + tRNA
+ GTP - tRNA
Input
Wash
Elution
SDS PAGE CBB staining
Urea PAGE Et-Br staining
tRNA
C
50mM NaCl
His6-CdiA-CT (H281A) + tRNA + GTP
-
+EF-Tu
+EF-Tu +EF-Ts
+EF-Tu +EF-TsΔCC
TsΔCC
150mM NaCl
D
+EF-Tu-Ts
+EF-Tu-TsΔCC
Tu-Ts
Tu-TsΔCC

Supplementary Figure S10: Interaction between CdiA-CT^{EC869} and Tu, Ts, and tRNA.

(**A**) Histidine-tagged CdiA-CT^{EC869} (5 μM, His$_6$-CdiA-CT) was mixed with equal amounts of Tu and Ts (and CdiI^{EC869}) in 200 μl of A-buffer, containing 20 mM Tris-Cl, pH 7.0, 150 mM NaCl, 5 mM β-mercaptoethanol, and 20 mM imidazole (input), and the mixture was loaded onto Ni-NTA resin (150 μl) in a Poly-Prep chromatography column (Bio-Rad, Japan). The flow-through (FT) was collected, and the Ni-NTA was washed three times with 500 μl of A-buffer (washes 1, 2, and 3). His$_6$-CdiA-CT was eluted from the resin with 200 μl of buffer, containing 20 mM Tris-Cl, pH 7.0, 150 mM NaCl, 5 mM β-mercaptoethanol, and 500 mM imidazole (elution). The samples were separated by 15% (w/v) SDS-PAGE and the gel was stained with Coomassie Brilliant Blue. (**B**) His-tagged CdiA-CT(H281A) was mixed with Tu and Ts in the presence or absence of tRNA or GTP, and the mixture was loaded onto a Ni-NTA column. The column was washed, and finally, CdiA-CT was eluted from the column. Proteins were visualized by SDS-PAGE and staining with CBB, and tRNA was visualized by PAGE and staining with ethidium bromide as in Figure 5G. (**C**) His-tagged CdiA-CT(H281A) was mixed with Tu or Tu and Ts (or Ts_ΔCC) in the presence of tRNA and GTP under two different salt conditions (upper panel: 50 mM NaCl and lower panel: 150 mM NaCl), and the mixture was loaded onto a Ni-NTA column. The column was washed, and finally, CdiA-CT was eluted from the column as in (B). (**D**) His-tagged CdiA-CT(H281A) was mixed with sg-Tu-Ts (or sgEF-Tu-TsΔCC) in the presence of tRNA and GTP. The column was washed, and finally, CdiA-CT was eluted from the column as in (B). For the experiments in (B)-(D), we used catalytically inactive CdiA-CT with H281A mutation, CdiA-CT (H281A), instead of using wild-type CdiA-CT, because tRNA could be cleaved during the pre-incubation of tRNA with wild-type CdiA-CT.

Figure S11

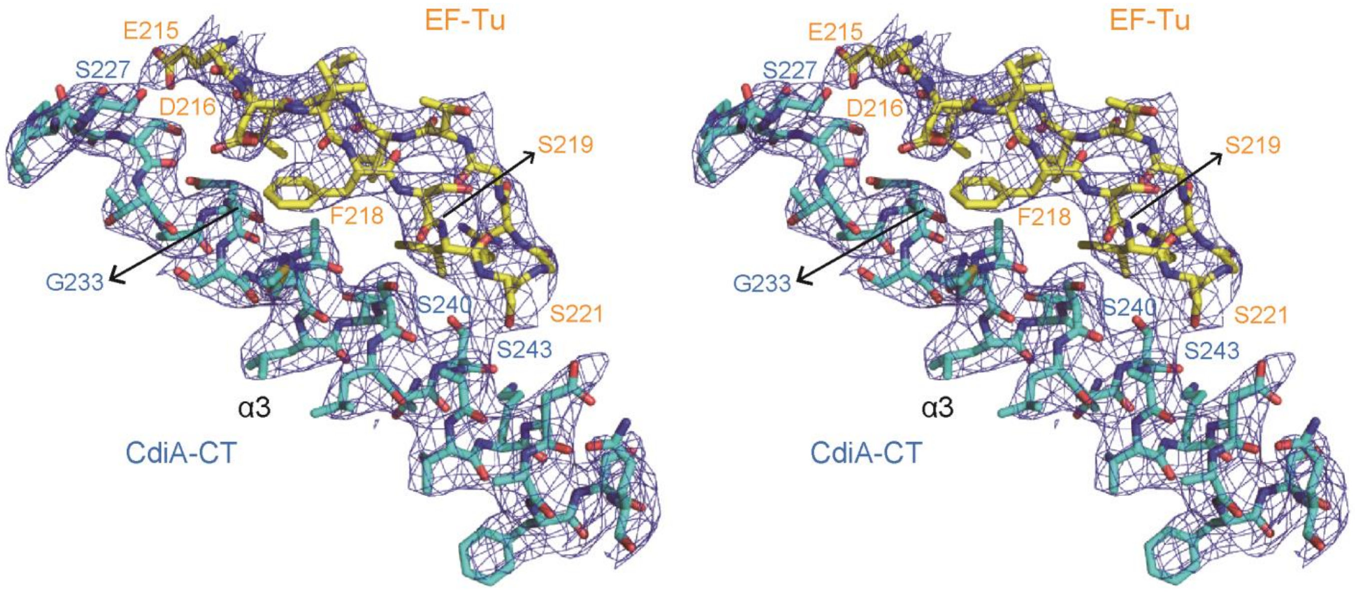

Supplementary Figure S11: Electron density map.

Stereoviews of the 2m*F*o-D*F*c electron densities of the residues around the CdiA-CT and Tu interface are shown in blue contoured at 1.0 σ.

Figure S12

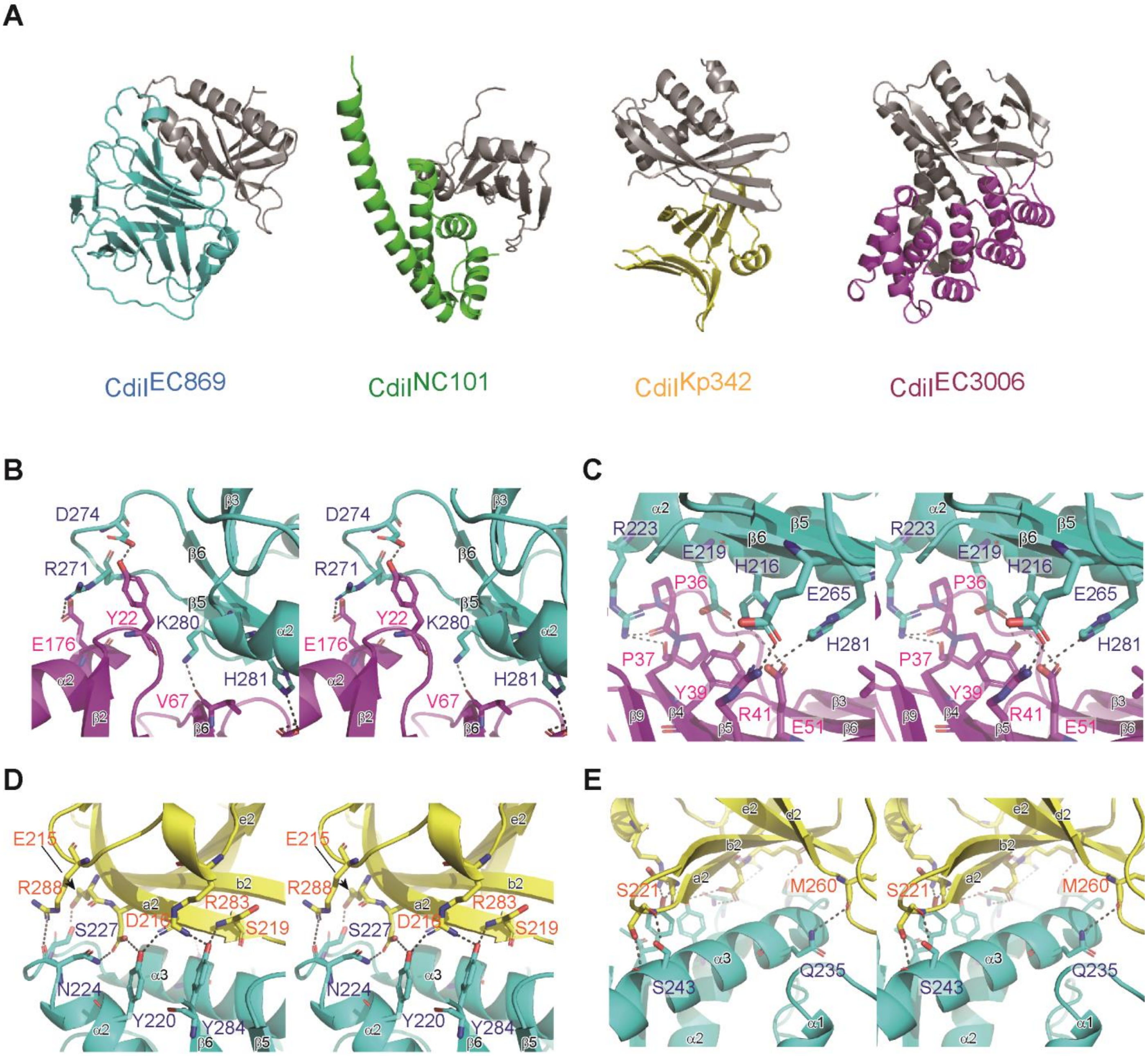

Supplementary Figure S12: (A) Structural comparison of CdiA-CT:CdiI complexes from *E. coli* EC869 (cyan), NC101 (green) (4), *Klebsiella pneumoniae* 342 (yellow) (5), and *E. coli* EC3006 (magenta) (5). For clarity, CdiA-CTs are colored in gray. (**B, C**) Interactions between CdiA-CT$^{\text{EC869}}$ (cyan) and CdiI$^{\text{EC869}}$ (magenta). (**D**, **E**) Interactions between CdiA-CT$^{\text{EC869}}$ (cyan) and domain II of Tu (yellow). Tu secondary structure elements are labeled according to the literature (6).

Figure S13

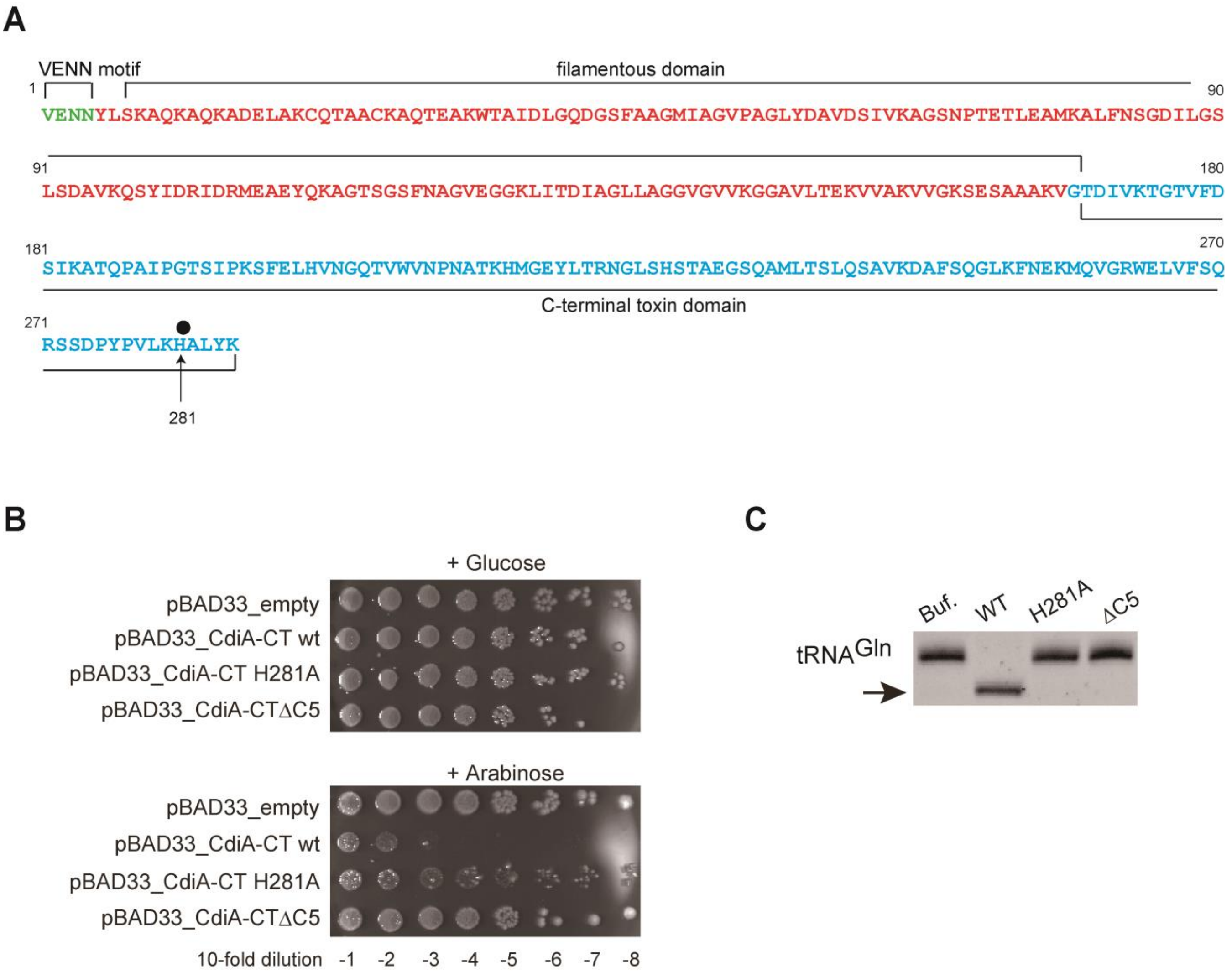

Supplementary Figure S13: CdiA-CT^{EC869} variants with reduced toxicity and tRNase activity.

(A) Amino acid sequence of CdiA-CT^{EC869}. The VENN motif (green), filamentous domain (red) and toxin domain (cyan) of CdiA-CT^{EC869} are depicted. **(B)** The cytotoxic activity of CdiA-CT^{EC869} is reduced by the His281Ala mutation (H218A) or deletion of five C-terminal amino acid residues (ΔC5). Overnight cultures of *E. coli* MG1655 transformed with pBAD33_CdiA-CT^{EC869}, pBAD33_CdiA-CT^{EC869}_H281A, and pBAD33_CdiA-CT^{EC869}_ ΔC5 were serially diluted, and the dilutions were spotted on LB agar plates containing 50 μg/ml chloramphenicol supplemented with 1% (w/v) arabinose (lower panel) or 1% (w/v) glucose (upper panel). **(C)** The His281Ala mutation (H281A) or deletion of the C-terminal five amino acids (ΔC5) decreased tRNA cleavage activity *in vitro*. Reactions were performed in the presence of Tu, Ts, and GTP, and the tRNA^{Gln} transcript was used as the substrate. The tRNA^{Gln} transcript (1.0 μM) was incubated at 37°C for 10 min with 0.5 μM of CdiA-CT^{EC869} or its variants in the presence of Tu (0.5 μM), Ts (0.5 μM), and GTP (1 mM). The arrow indicates tRNA^{Gln} cleaved by CdiA-CT^{EC869}.

Supplementary Table S1

Nucleotide sequences of the synthetic *E. coli* tRNAs and CdiA-CT/CdiI^{EC869} genes

Gene	Nucleotide sequence
tRNA^{Gln} U1A72 (wt)	5'-<br>gaattcTAATACGACTCACTATAGGGAGACCACAACGGTTTCCCTCTAGATGG<br>GGTATCGCCAAGCGGTAAGGCACCGGATTCTGATTCCGGCATTCCGAGGTT<br>CGAATCCTCGTACCCCAGCCAggctagctaCATCCAAGCTT-3'
tRNA^{Gln}U1G72	5'-<br>gaattcTAATACGACTCACTATAGGGAGACCACAACGGTTTCCCTCTAGATGG<br>GGTATCGCCAAGCGGTAAGGCACCGGATTCTGATTCCGGCATTCCGAGGTT<br>CGAATCCTCGTACCCCGGCCAggctagctaCATCCAAGCTT-3'
tRNA^{Gln}U1C72	5'-<br>gaattcTAATACGACTCACTATAGGGAGACCACAACGGTTTCCCTCTAGATGG<br>GGTATCGCCAAGCGGTAAGGCACCGGATTCTGATTCCGGCATTCCGAGGTT<br>CGAATCCTCGTACCCCCGCCAggctagctaCATCCAAGCTT-3'
tRNA^{Gln}U1U72	5'-<br>gaattcTAATACGACTCACTATAGGGAGACCACAACGGTTTCCCTCTAGATGG<br>GGTATCGCCAAGCGGTAAGGCACCGGATTCTGATTCCGGCATTCCGAGGTT<br>CGAATCCTCGTACCCCTGCCAggctagctaCATCCAAGCTT-3'
tRNA^{Gln}A1A72	5'-<br>gaattcTAATACGACTCACTATAGGGAGACCACAACGGTTTCCCTCTAGAAGG<br>GGTATCGCCAAGCGGTAAGGCACCGGATTCTGATTCCGGCATTCCGAGGTT<br>CGAATCCTCGTACCCCAGCCAggctagctaCATCCAAGCTT-3'
tRNA^{Gln}G1A72	5'-<br>gaattcTAATACGACTCACTATAGGGAGACCACAACGGTTTCCCTCTAGAGGG<br>GGTATCGCCAAGCGGTAAGGCACCGGATTCTGATTCCGGCATTCCGAGGTT<br>CGAATCCTCGTACCCCAGCCAggctagctaCATCCAAGCTT-3'
tRNA^{Gln}C1A72	5'-<br>gaattcTAATACGACTCACTATAGGGAGACCACAACGGTTTCCCTCTAGACGG<br>GGTATCGCCAAGCGGTAAGGCACCGGATTCTGATTCCGGCATTCCGAGGTT<br>CGAATCCTCGTACCCCAGCCAggctagctaCATCCAAGCTT-3'
tRNA^{Gln}A1U72	5'-<br>gaattcTAATACGACTCACTATAGGGAGACCACAACGGTTTCCCTCTAGAAGG<br>GGTATCGCCAAGCGGTAAGGCACCGGATTCTGATTCCGGCATTCCGAGGTT<br>CGAATCCTCGTACCCCTGCCAggctagctaCATCCAAGCTT-3'
tRNA^{Gln}G1C72	5'-<br>gaattcTAATACGACTCACTATAGGGAGACCACAACGGTTTCCCTCTAGAGGG

	GGTATCGCCAAGCGGTAAGGCACCGGATTCTGATTCCGGCATTCCGAGGTT CGAATCCTCGTACCCCCGCCAggctagctaCATCCAAGCTT-3'
tRNA^{Gln}A73	5'- gaattcTAATACGACTCACTATAGGGAGACCACAACGGTTTCCCTCTAGATGG GGTATCGCCAAGCGGTAAGGCACCGGATTCTGATTCCGGCATTCCGAGGTT CGAATCCTCGTACCCCAACCAggctagctaCATCCAAGCTT-3'
tRNA^{Gln}U73	5'- gaattcTAATACGACTCACTATAGGGAGACCACAACGGTTTCCCTCTAGATGG GGTATCGCCAAGCGGTAAGGCACCGGATTCTGATTCCGGCATTCCGAGGTT CGAATCCTCGTACCCCATCCAggctagctaCATCCAAGCTT-3'
tRNA^{Trp}	5'- GAATTCTAATACGACTCACTATAGGGAGACCACAACGGTTTCCCTCTAGAA GGGGCGTAGTTCAATTGGTAGAGCACCGGTCTCCAAAACCGGGTGTTGGGA GTTCGAGTCTCTCCGCCCCTGCCAGGCTAGCTACATCCAAGCTT-3'
CdiA-CT^{EC869} (amino acid residues 1–286) and CdiI^{EC869} (blue letters; amino acid residues 1–179) coding sequences	5'- GTTGAGAATAACTATCTGAGTAAAGCCCAGAAAGCTCAAAAAGCCGATGA GCTGGCTAAATGTCAGACTGCTGCCTGTAAGGCTCAGACAGAAGCAAAAT GGACGGCAATTGACCTGGGGCAGGATGGTAGCTTTGCTGCAGGAATGATTG CTGGGGTTCCTGCCGGGCTGTATGATGCTGTGGATAGTATTGTAAAAGCAG GTTCTAACCCGACGGAAACCCTGGAGGCAATGAAAGCGCTGTTTAACAGC GGCGATATCTTGGGTTCGTTATCGGATGCGGTTAAGCAATCTTATATAGAC CGTATTGACCGAATGGAAGCCGAATATCAGAAAGCTGGTACCAGCGGTTC GTTTAATGCAGGCGTTGAAGGCGGTAAGCTGATTACTGATATTGCCGGGCT GCTGGCTGGCGGCGTTGGCGTTGTGAAAGGTGGTGCGGTACTGACGGAGA AGGTTGTTGCTAAGGTTGTGGGTAAGTCTGAATCAGCAGCGGCAAAAGTTG GTACAGATATTGTTAAAACAGGAACCGTTTTCGACTCAATTAAAGCGACTC AACCTGCGATACCAGGAACATCCATTCCAAAATCTTTTGAATTACATGTTA ACGGACAAACCGTTTGGGTAAACCCAAATGCAACTAAACATATGGGCGAA TATTTAACACGGAATGGATTGTCTCACAGTACAGCAGAAGGAAGCCAAGC CATGCTGACCAGTCTTCAAAGTGCGGTTAAAGATGCATTTTCGCAGGGATT AAAATTTAACGAAAAAATGCAAGTTGGACGCTGGGAACTGGTATTTAGCC AACGGTCTTCAGATCCTTATCCAGTATTAAAGCATGCGTTGTATAAATAAT GAAATTAACTGTAGATAGCGTTATTAATGAACCTAGAAGCGTAGCCATTAC TATTGATGGTTATATTCCCGTTGATATAAAGATTATTGATTCTAAAAAGCTT CCGCCTTTGTATTGGCGGGGCGGGGATGGCAAAAAAAACCTACTTGAACTG GCTGTACTACCAGAAAATGGTTTTTTATCATCCATCACATTGGTAATGATAG CATCAGACTCAATTCATAAAACAGACTCCTTGTCAGTATCTTTACCAAGCA GTGAGTGTGGAGTTCCTGTAGTGAATACAAAACTGTGGAGCCACTCAGAAA GTGATGATTTTAGTCGTCGTTTTGTTGACGATTTTAGCCTTGATATTGAGGT GATTATATCATCAGAGTCTATGTTATTAACGATTGGAGAGAATAAAAAGGT

	AACTAGCTGGATAAAATGTAGCGATAATTTTTATCTCGGGATAGATGCAGGAAGAAATGTCGTTCATTTGTATTTGGATAAGTTAACACCAAGTGAAGTGGAAAGTTTTTTTGAGGCAGTAGGTTAG-3'

Nucleotide sequences of Tu and Ts

Tu (amino acid residues 1–394) coding sequence	5'-TCTAAAGAAAAGTTTGAACGTACAAAACCGCACGTTAACGTCGGTACTATCGGCCACGTTGACCATGGTAAAACAACGCTGACCGCTGCAATCACTACCGTACTGGCTAAAACCTACGGCGGTGCTGCTCGCGCATTCGACCAGATCGATAACGCGCCGGAAGAAAAAGCTCGTGGTATCACCATCAACACTTCTCACGTTGAATACGACACCCCGACCCGTCACTACGCACACGTAGACTGCCCGGGGCACGCCGACTATGTTAAAAACATGATCACCGGTGCTGCGCAGATGGACGGCGCGATCCTGGTAGTTGCTGCGACTGACGGCCCGATGCCGCAGACTCGTGAGCACATCCTGCTGGGTCGTCAGGTAGGCGTTCCGTACATCATCGTGTTCCTGAACAAATGCGACATGGTTGATGACGAAGAGCTGCTGGAACTGGTTGAAATGGAAGTTCGTGAACTTCTGTCTCAGTACGACTTCCCGGGCGACGACACTCCGATCGTTCGTGGTTCTGCTCTGAAAGCGCTGGAAGGCGACGCAGAGTGGGAAGCGAAAATCCTGGAACTGGCTGGCTTCCTGGATTCTTACATTCCGGAACCAGAGCGTGCGATTGACAAGCCGTTCCTGCTGCCGATCGAAGACGTATTCTCCATCTCCGGTCGTGGTACCGTTGTTACCGGTCGTGTAGAACGCGGTATCATCAAAGTTGGTGAAGAAGTTGAAATCGTTGGTATCAAAGAGACTCAGAAGTCTACCTGTACTGGCGTTGAAATGTTCCGCAAACTGCTGGACGAAGGCCGTGCTGGTGAGAACGTAGGTGTTCTGCTGCGTGGTATCAAACGTGAAGAAATCGAACGTGGTCAGGTACTGGCTAAGCCGGGCACCATCAAGCCGCACACCAAGTTCGAATCTGAAGTGTACATTCTGTCCAAAGATGAAGGCGGCCGTCATACTCCGTTCTTCAAAGGCTACCGTCCGCAGTTCTACTTCCGTACTACTGACGTGACTGGTACCATCGAACTGCCGGAAGGCGTAGAGATGGTAATGCCGGGCGACAACATCAAAATGGTTGTTACCCTGATCCACCCGATCGCGATGGACGACGGTCTGCGTTTCGCAATCCGTGAAGGCGGCCGTACCGTTGGCGCGGGCGTTGTAGCAAAAGTTCTGAGCTAA-3'
Ts (amino acid residues 1–283) coding sequence	5'-GCTGAAATTACCGCATCCCTGGTAAAAGAGCTGCGTGAGCGTACTGGCGCAGGCATGATGGATTGCAAAAAAGCACTGACTGAAGCTAACGGCGACATCGAGCTGGCAATCGAAAACATGCGTAAGTCCGGTGCTATTAAAGCAGCGAAAAAAGCAGGCAACGTTGCTGCTGACGGCGTGATCAAAACCAAAATCGACGGCAACTACGGCATCATTCTGGAAGTTAACTGCCAGACTGACTTCGTTGCAAAAGACGCTGGTTTCCAGGCGTTCGCAGACAAAGTTCTGGACGCAGCTGTTGCTGGCAAAATCACTGACGTTGAAGTTCTGAAAGCACAGTTCGAAGAAGAACGTGTTGCGCTGGTAGCGAAAATTGGTGAAAACATCAACATTC

	GCCGCGTTGCTGCGCTGGAAGGCGACGTTCTGGGTTCTTATCAGCACGGT GCGCGTATCGGCGTTCTGGTTGCTGCTAAAGGCGCTGACGAAGAGCTGGT TAAACACATCGCTATGCACGTTGCTGCAAGCAAGCCAGAATTCATCAAA CCGGAAGACGTATCCGCTGAAGTGGTAGAAAAAGAATACCAGGTACAGC TGGATATCGCGATGCAGTCTGGTAAGCCGAAAGAAATCGCAGAGAAAAT GGTTGAAGGCCGCATGAAGAAATTCACCGGCGAAGTTTCTCTGACCGGT CAGCCGTTCGTTATGGAACCAAGCAAAACTGTTGGTCAGCTGCTGAAAG AGCATAACGCTGAAGTGACTGGCTTCATCCGCTTCGAAGTGGGTGAAGG CATCGAGAAAGTTGAGACTGACTTTACAGCAGAAGTTGCTGCGATGTCC AAGCAGTCTTGA-3'

Supplementary Table S2: Oligonucleotide sequences

Oligonucleotide	Sequence (5′ to 3′ direction)
GlnRS-for_NdeI	AGCTAGCTCATATGAGTGAGGCAGAAGCCCG
GlnRS-rev_XhoI	AGCTCTCGAGCTCGCCTACTTTCGCCCAGGTA
Tu_N51A-for	gcgGCGCCGGAAGAAAAAGCTCGTGGTATCACC
Tu_N51A-rev	ATCGATCTGGTCGAATGCGCGAGCAGCACC
Tu_E54A-for	gcgGAAAAAGCTCGTGGTATCACCATCAACACTTC
Tu_E54A-rev	CGGCGCGTTATCGATCTGGTCGAATGCGCGAGC
Tu_R58A-for	gcgGGTATCACCATCAACACTTCTCACGTTG
Tu_R58A-rev	AGCTTTTTCTTCCGGCGCGTTATCGATCTGGTCG
Tu_H66A-for	gcgGTTGAATACGACACCCCGACCCGTCACTACG
Tu_H66A-rev	AGAAGTGTTGATGGTGATACCACGAGCTTTTTC
Tu_H84A-for	gcgGCCGACTATGTTAAAAACATGATCACCGG
Tu_H84A-rev	CCCCGGGCAGTCTACGTGTGCGTAGTGACGGGTC
Tu_Y87A-for	gcgGTTAAAAACATGATCACCGGTGCTGCGCAGA
Tu_Y87A-rev	GTCGGCGTGCCCCGGGCAGTCTACGTGTGC
Tu_K89A N90A-for	gcggcgATGATCACCGGTGCTGCGCAGATGGAC
Tu_K89A N90A-rev	AACATAGTCGGCGTGCCCCGGGCAGTCTACGTG
Tu_N135A-for	gcgAAATGCGACATGGTTGATGACGAAGAGCTGC
Tu_N135A-rev	CAGGAACACGATGATGTACGGAACGCCTACCTG
Tu_F218A-for	gcgTCCATCTCCGGTCGTGGTACCGTTGTTACCGG
Tu_F218A-rev	TACGTCTTCGATCGGCAGCAGGAACGGCTTG
Tu_R223A-for	gcgGGTACCGTTGTTACCGGTCGTGTAGAACGCGG
Tu_R223A-rev	ACCGGAGATGGAGAATACGTCTTCGATCGGCAGC
Tu_E259A-for	gcgATGTTCCGCAAACTGCTGGACGAAGGCCG
Tu_E259A-rev	AACGCCAGTACAGGTAGACTTCTGAGTCTCTTTG
Tu_R262A-for	gcgAAACTGCTGGACGAAGGCCGTGCTGGTGAG
Tu_R262A-rev	GAACATTTCAACGCCAGTACAGGTAGACTTCTGAG
Tu_R283A-for	gcgGAAGAAATCGAACGTGGTCAGGTACTGGC
Tu_R283A-rev	TTTGATACCACGCAGCAGAACACCTACGTTCTC
Tu_R288A-for	gcgGGTCAGGTACTGGCTAAGCCGGGCACCATC
Tu_R288A-rev	TTCGATTTCTTCACGTTTGATACCACGCAGCAG
Tu_R318A H319A-for	gcggcgACTCCGTTCTTCAAAGGCTACCGTCCGC
Tu_R319A H319A-rev	GCCGCCTTCATCTTTGGACAGAATGTACACTTCAG
Tu_T320A-for	gcgCCGTTCTTCAAAGGCTACCGTCCGCAG
Tu_T320A-rev	ATGACGGCCGCCTTCATCTTTGGACAGAATGTAC
Ts_A202E-for	gaaATGCAGTCTGGTAAGCCGAAAGAAATC
Ts_A202E-rev	GATATCCAGCTGTACCTGGTATTCTTTTTCTACC

Ts_K207A K209A-for	gcgCCGgcgGAAATCGCAGAGAAAATGGTTGAAGGC
Ts_K207A K209A-rev	ACCAGACTGCATCGCGATATCCAGCTGTACC
Ts_K214E-for	gaaATGGTTGAAGGCCGCATGAAGAAATTCACC
Ts_K214E-rev	CTCTGCGATTTCTTTCGGCTTACCAGACTGCATC
Ts_R219P-for	ccgATGAAGAAATTCACCGGCGAAGTTTCTCTG
Ts_R219P-rev	GCCTTCAACCATTTTCTCTGCGATTTCTTTCGG
Ts_R219A K221A K222A-for	gcgATGgcggcgTTCACCGGCGAAGTTTCTCTG
Ts_R219A K221A K222A-rev	GCCTTCAACCATTTTCTCTGCGATTTCTTTCGG
CdiA-for_NdeI	AGCTAGCTCATATGGTTGAGAATAACTATCTGAGTAAAGCC
CdiA-rev_XhoI_HindIII	AGCTAAGCTTCTCGAGTTATTTATACAACGCATGCTTTAATACTGG
CdiI-for_NdeI	AGCTAGCTCATATGAAATTAACTGTAGATAGCGTT
CdiI-rev_XhoI	AGCTCTCGAGCTAACCTACTGCCTCAAA
CdiA_H281A-for	gctGCGTTGTATAAATAATGAAATTAACTG
CdiA_H281A-rev	CTTTAATACTGGATAAGGATCTG
CdiAΔC5-rev_XhoI_HindIII	AGCTAAGCTTCTCGAGTTACTTTAATACTGGATAAGGATCTGAAGACCG
CdiA_H216A-for	GCGATGGGCGAATATTTAACACGGAATGG
CdiA_H216A-rev	TTTAGTTGCATTTGGGTTTACCCAAACGG
CdiA_Y220F-for	TTTTTAACACGGAATGGATTGTCTCACAG
CdiA_Y220F-rev	TTCGCCCATGTGTTTAGTTGCATTTGGG
CdiA_R263A-for	gccTGGGAACTGGTATTTAGCCAACGG
CdiA_R263A-rev	TCCAACTTGCATTTTTTCGTTAAATTTTAATCC
CdiA_R271A-for	gcgTCTTCAGATCCTTATCCAGTATTAAAG
CdiA_R271A-rev	TTGGCTAAATACCAGTTCCCAGCGTCC
Ala_probe	TGGAGCTAAGCGGGATCGAACCGCTGaCCTCTTGC
Asn_probe	CTCCTCTGACTGGACTCGAACCAGTGaCATACGGA
Gln_probe	CTGGGGTACGAGGATTCGAACCTCGGaATGCCGGA
Met_probe	TGGCTACGACGGGATTCGAACCTGTGACCCCATCA
fMet_probe	TTGCGGGGGCCGGATTTGAACCGACGaCCTTCGGG
Trp_probe	CAGGGGCGGAGAGACTCGAACTCCCAaCACCCGGT
Infusion_sumo_TsTu_For	aacagattggtggtCATATGGCTGAAATTACCGCATCCCTG
Infusion_sumo_TsTu_Rev	ccctcgagcccgggCATATGTTAGCTCAGAACTTTTGCTACAACG

Supplementary References

1. Suzuki, T., Terasaki, M., Takemoto-Hori, C., Hanada, T., Ueda, T., Wada, A. and Watanabe, K. (2001) Proteomic Analysis of the Mammalian Mitochondrial Ribosome: IDENTIFICATION OF PROTEIN COMPONENTS IN THE 28 S SMALL SUBUNIT*. *Journal of Biological Chemistry*, **276**, 33181-33195.
2. Chan, P.P. and Lowe, T.M. (2015) GtRNAdb 2.0: an expanded database of transfer RNA genes identified in complete and draft genomes. *Nucleic Acids Res.*, **44**, D184-D189.
3. Wieden, H.-J., Gromadski, K., Rodnin, D. and Rodnina, M.V. (2002) Mechanism of Elongation Factor (EF)-Ts-catalyzed Nucleotide Exchange in EF-Tu: CONTRIBUTION OF CONTACTS AT THE GUANINE BASE*. *Journal of Biological Chemistry*, **277**, 6032-6036.
4. Michalska, K., Gucinski, G.C., Garza-Sanchez, F., Johnson, P.M., Stols, L.M., Eschenfeldt, W.H., Babnigg, G., Low, D.A., Goulding, C.W., Joachimiak, A. *et al.* (2017) Structure of a novel antibacterial toxin that exploits elongation factor Tu to cleave specific transfer RNAs. *Nucleic Acids Res*, **45**, 10306-10320.
5. Gucinski, G.C., Michalska, K., Garza-Sánchez, F., Eschenfeldt, W.H., Stols, L., Nguyen, J.Y., Goulding, C.W., Joachimiak, A. and Hayes, C.S. (2019) Convergent Evolution of the Barnase/EndoU/Colicin/RelE (BECR) Fold in Antibacterial tRNase Toxins. *Structure (London, England : 1993)*, **27**, 1660-1674.e1665.
6. Nissen, P., Thirup, S., Kjeldgaard, M. and Nyborg, J. (1999) The crystal structure of Cys-tRNACys-EF-Tu-GDPNP reveals general and specific features in the ternary complex and in tRNA. *Structure*, **7**, 143-156.
